# Apoplastic Hydrogen Peroxide in the Growth Zone of the Maize Primary Root. Increased Levels Differentially Modulate Root Elongation Under Well-Watered and Water-Stressed Conditions

**DOI:** 10.3389/fpls.2020.00392

**Published:** 2020-04-21

**Authors:** Priya Voothuluru, Pirjo Mäkelä, Jinming Zhu, Mineo Yamaguchi, In-Jeong Cho, Melvin J. Oliver, John Simmonds, Robert E. Sharp

**Affiliations:** ^1^Division of Plant Sciences, University of Missouri, Columbia, MO, United States; ^2^Interdisciplinary Plant Group, University of Missouri, Columbia, MO, United States; ^3^Department of Agricultural Sciences, University of Helsinki, Helsinki, Finland; ^4^United States Department of Agriculture-Agricultural Research Service, Plant Genetics Research Unit, University of Missouri, Columbia, MO, United States; ^5^Agriculture and Agri-Food Canada, Ottawa, ON, Canada

**Keywords:** cell elongation, cell production, root growth, hydrogen peroxide, kinematics, reactive oxygen species, water stress, *Zea mays*

## Abstract

Reactive oxygen species (ROS) can act as signaling molecules involved in the acclimation of plants to various abiotic and biotic stresses. However, it is not clear how the generalized increases in ROS and downstream signaling events that occur in response to stressful conditions are coordinated to modify plant growth and development. Previous studies of maize (*Zea mays* L.) primary root growth under water deficit stress showed that cell elongation is maintained in the apical region of the growth zone but progressively inhibited further from the apex, and that the rate of cell production is also decreased. It was observed that apoplastic ROS, particularly hydrogen peroxide (H_2_O_2_), increased specifically in the apical region of the growth zone under water stress, resulting at least partly from increased oxalate oxidase activity in this region. To assess the function of the increase in apoplastic H_2_O_2_ in root growth regulation, transgenic maize lines constitutively expressing a wheat *oxalate oxidase* were utilized in combination with kinematic growth analysis to examine effects of increased apoplastic H_2_O_2_ on the spatial pattern of cell elongation and on cell production in well-watered and water-stressed roots. Effects of H_2_O_2_ removal (via scavenger pretreatment) specifically from the apical region of the growth zone were also assessed. The results show that apoplastic H_2_O_2_ positively modulates cell production and root elongation under well-watered conditions, whereas the normal increase in apoplastic H_2_O_2_ in water-stressed roots is causally related to down-regulation of cell production and root growth inhibition. The effects on cell production were accompanied by changes in spatial profiles of cell elongation and in the length of the growth zone. However, effects on overall cell elongation, as reflected in final cell lengths, were minor. These results reveal a fundamental role of apoplastic H_2_O_2_ in regulating cell production and root elongation in both well-watered and water-stressed conditions.

## Introduction

The growth of plant organs does not occur indiscriminately but is restricted in its distribution to certain regions that are referred to as the growth zones. Within these zones, there is considerable spatial and temporal heterogeneity of cell production and cell expansion rates (Erickson and Silk, 1980). This heterogeneity can occur during the course of development (Beemster and Baskin, 1998) and in response to various environmental conditions (Sharp et al., 1988; Sacks et al., 1997; Muller et al., 1998; Yang et al., 2017), and thereby impacts the structure and function of different plant tissues and organs.

Drought is the major environmental factor reducing plant growth and crop productivity on a global basis (Boyer, 1982; Boyer et al., 2013). Understanding how water-stressed plants regulate growth and development of different organs is vitally important for developing crops with improved drought tolerance (Sharp and Davies, 1989; Gonzalez et al., 2012; Tardieu et al., 2018). Maintenance of root system development is a prominent adaptation of plants to water deficit (Ober and Sharp, 2007), which can enable access to water from deeper soil profiles (Sharp and Davies, 1985; Sponchiado et al., 1989; Kirkegaard et al., 2007). In some circumstances, to reach moist soil, roots must grow through soil that is already dry, and it has been demonstrated that some root types, including the primary root of several species, can continue growing at low soil water potentials that completely inhibit shoot growth (Sharp and Davies, 1979; Westgate and Boyer, 1985; Sharp et al., 1988; Spollen et al., 1993; Yamaguchi et al., 2010).

The physiological mechanisms underlying root growth maintenance at low water potentials have been studied extensively in the primary root of maize (*Zea mays* L.; reviewed in Sharp et al., 2004; Yamaguchi and Sharp, 2010; Ober and Sharp, 2013). Kinematic growth analysis (Erickson and Silk, 1980; Walter et al., 2009) was used to characterize the spatial and temporal patterns of cell expansion within the growth zone (Sharp et al., 1988; Liang et al., 1997). The results demonstrated that cell elongation is differentially responsive to water stress in different regions. Local elongation rates are maintained in the apical region even under conditions of severe water stress (water potential of −1.6 MPa), but are then progressively inhibited as cells are displaced further from the apex, resulting in a shortened growth zone. Interestingly, despite the maintenance of cell elongation in the apical region, which encompasses the meristem, the cell production rate was reported to decrease by 30% or more in maize primary roots growing under water stress (Fraser et al., 1990; Saab et al., 1992; Sacks et al., 1997). It is unclear whether the decrease in cell production reflects a negative effect of water stress or, potentially, a component of root growth adaptation to water-limited conditions (Sacks et al., 1997). Mechanisms regulating the decrease in cell production in water-stressed roots have not been investigated.

In association with the spatially variable response of cell elongation to water stress in the maize primary root, cell wall extension properties are enhanced in the apical region of growth maintenance but reduced in the basal region of growth inhibition (Wu et al., 1996). The increase in extensibility in the apical region helps to maintain cell elongation despite incomplete turgor maintenance (Spollen and Sharp, 1991). Integration of spatial growth analyses with functional genomics revealed that the majority of changes involved region-specific patterns of responses (Zhu et al., 2007; Spollen et al., 2008; Voothuluru et al., 2016). Transcriptome and cell wall proteome analyses showed that gene expression and abundance of proteins involved in generating reactive oxygen species (ROS) increased under water stress, particularly in the apical region (Zhu et al., 2007; Spollen et al., 2008). Subsequent studies confirmed that apoplastic hydrogen peroxide (H_2_O_2_) increased specifically in the apical region of the growth zone in water-stressed compared with well-watered roots (Voothuluru and Sharp, 2013).

Apoplastic ROS may have cell wall loosening or tightening effects that could be region specific, and may also have other growth regulatory functions (Córdoba-Pedregosa et al., 2003; Foreman et al., 2003; Tyburski et al., 2010). Apoplastic ROS have been implicated in cleaving cell wall polysaccharides to promote wall loosening and cell expansion (Schopfer, 2001; Fry, 2004; Müller et al., 2009). Schopfer (2001) provided evidence that hydroxyl radicals are involved in wall loosening and growth promotion in maize coleoptiles, and inhibition of hydroxyl radical production using various ROS scavengers or inhibitors of ROS-producing enzymes resulted in growth inhibition of maize primary roots (Liszkay et al., 2004) and leaves (Rodriguez et al., 2002) under well-watered conditions. On the other hand, apoplastic ROS could potentially be involved in signaling processes that regulate cell production (Menon and Goswami, 2007; Foyer and Noctor, 2011). An increase in apoplastic ROS production and perception has been implicated in cellular signaling and the regulation of nuclear gene transcription (Padmanabhan and Dinesh-Kumar, 2010; Shapiguzov et al., 2012), and studies in both animal and plant systems suggested that redox balance is critical for cell cycle progression and the maintenance of cell proliferation (Burhans and Heintz, 2009; Tsukagoshi et al., 2010; Yu et al., 2013; Tsukagoshi, 2016). Whether the increase in apoplastic ROS in the apical region of the growth zone of water-stressed roots is involved in regulating wall loosening and/or cell production has not been investigated.

In plants, apoplastic ROS can be generated by different enzymatic and non-enzymatic processes. The NADPH oxidases, located in the plasma membrane, utilize cytosolic NADPH to generate apoplastic superoxide (Foreman et al., 2003; Liszkay et al., 2004). The superoxide could participate in signaling processes in the apoplast or can be converted into H_2_O_2_ or hydroxyl radicals by action of superoxide dismutases and peroxidases (Liszkay et al., 2004; Dunand et al., 2007). Apoplastic H_2_O_2_ can also be produced by cell wall-localized enzymes including oxalate oxidases and polyamine oxidases (Davidson et al., 2009; Waszczak et al., 2018) or by the non-enzymatic degradation of apoplastic ascorbate (Green and Fry, 2005). The increase in apoplastic ROS in the growth zone of water-stressed roots likely results, at least partly, from the marked increase in gene expression, protein abundance and activity of oxalate oxidase that occurs in this region (Zhu et al., 2007; Spollen et al., 2008; Voothuluru and Sharp, 2013). Oxalate oxidases catalyze the conversion of oxalate to H_2_O_2_ and CO_2_, and are known to be cell wall localized (Davidson et al., 2009). In this study, transgenic maize lines constitutively expressing a wheat *oxalate oxidase* (Ramputh et al., 2002; Mao et al., 2007) were utilized in combination with kinematic growth analysis to examine effects of increased oxalate oxidase activity and apoplastic H_2_O_2_ on the spatial patterns of elongation and on cell production rates in well-watered and water-stressed maize primary roots. The results indicate that apoplastic H_2_O_2_ positively modulates cell production and root elongation under well-watered conditions, whereas in water-stressed roots, increased apoplastic H_2_O_2_ is causally related to down-regulation of cell production and root growth inhibition. Although spatial growth patterns were altered, increased H_2_O_2_ levels had relatively minor effects on overall cell elongation, as reflected in final cell lengths, in roots growing under both well-watered and water-stressed conditions. Potential mechanisms by which apoplastic H_2_O_2_ may modulate cell production and root elongation are discussed.

## Materials and Methods

### Plant Materials and Growth Conditions

Transgenic maize (*Z. mays* L.) lines that were stably expressing a wheat *oxalate oxidase* gene regulated by a constitutive rice actin promoter were used. The lines were in the CK44 (Transgenic T_8_ generation) and B73 (T_5_ generation) inbred backgrounds (Ramputh et al., 2002; Mao et al., 2007). Corresponding segregated transgene negative lines were used as controls and are referred to as “wild-type.” Experiments focused on the CK44 line, and key findings were repeated with the B73 line.

Seeds were sterilized in 5% NaClO (v/v) for 15 min, rinsed with deionized water for 15 min, imbibed in aerated 1 mM CaSO_4_ for 24 h, and germinated between sheets of germination paper moistened with 1 mM CaSO_4_ at 29°C and near-saturation humidity in the dark. Seedlings with primary roots 10–20 mm in length were transplanted against the sides of Plexiglas boxes containing vermiculite (no. 2A, Therm-O-Rock East Inc.) at water potentials of −0.03 MPa (well-watered) or −1.6 MPa (water-stressed), which were obtained by thorough mixing with pre-calibrated amounts of 1 mM CaSO_4_ (Sharp et al., 1988; Spollen et al., 2000). In some experiments, vermiculite water potentials of −0.3 and −0.8 MPa were also used. Water potentials were measured in each experiment by isopiestic thermocouple psychrometry (Boyer and Knipling, 1965). Seedlings were grown at 29°C and near-saturation humidity in the dark (to minimize further drying of the media) until harvest (Sharp et al., 1988). Primary root elongation rates were determined by periodically marking the position of the root apices on the sides of the boxes. Transplanting, growth measurements and harvesting were performed using a green “safe” light (Saab et al., 1990).

### Oxalate Oxidase Activity Staining Assay

*In-situ* oxalate oxidase activity was detected in apical segments (approximately 12 mm in length; 48 and 72 h after transplanting) or in transverse sections (48 h after transplanting) of primary roots using a solution containing 25 mM succinic acid, 3.5 mM EDTA, 2.5 mM oxalic acid at pH 4, and 0.6 mg mL^–1^ 4-chloro-1-naphthol (Dumas et al., 1995; Voothuluru and Sharp, 2013). Segments were stained for 24 h at 25°C (length measurements showed that the segments did not grow during staining). Transverse sections (14 μm) were obtained using a cryostat microtome (Leica CM 1850) at 1–2, 5–6, and 9.5–10.5 mm from the apex, and stained for 45 min (Caliskan and Cuming, 1998). The segments or sections were then washed several times in deionized water and imaged using a stereomicroscope (Leica MZFLIII). Controls for oxalate oxidase staining were incubated in staining solution without oxalic acid. The oxalate oxidase activity in water-stressed roots was evaluated using non-isoosmotic staining solution, since it was previously shown that using melibiose to lower the water potential (as used in experiments described below) interferes with the assay (Voothuluru and Sharp, 2013).

### Oxalate Oxidase Transgene Expression

Primary roots were collected from batches of five to eight seedlings at 36 and 48 h after transplanting. Root tips were sectioned into apical (0–5 mm) and basal (5–12 mm) regions, frozen in liquid nitrogen and stored at −80°C. Samples were ground in liquid nitrogen and RNA was extracted using RNeasy (Qiagen). After cleaning with 0.5 units of DNase (Invitrogen) μg^–1^ total RNA, 5 μg RNA was used to synthesize single-stranded cDNA using one unit of Superscript II Reverse Transcriptase (Invitrogen) and 0.5 μg oligo dT primers. Transgene-specific primers (Forward 5′ CATGGTCGTCTCCTTCAACA and Reverse 3′ CATTTCAGGGAAGGCTCCTA) were designed using Primer3 Software, and 0.2 μg aliquots were used for qRT-PCR with 1 μg cDNA to amplify the wheat *oxalate oxidase* expression. Glyceraldehyde phosphate dehydrogenase was used as a reference gene.

### *In situ* Imaging of Apoplastic ROS

Imaging of apoplastic ROS in the apical region of the primary root was conducted using the fluorescent dye 2′,7′-dichlorodihydrofluorescein (H_2_DCF; custom synthesized by Molecular Probes), as described by Zhu et al. (2007). The dye is a derivative of 5-(and-6)-carboxy-2′,7′- H_2_DCF diacetate (carboxy-H_2_DCFDA, an indicator of intracellular ROS) in which the acetate groups (which allow the molecule to cross the plasma membrane) have been cleaved. Evidence of apoplastic localization of H_2_DCF staining in the apical region of well-watered and water-stressed maize primary roots was detailed in Zhu et al. (2007).

Staining was conducted using a protocol to minimize diffusion of the dye and ROS from the apoplast (Zhu et al., 2007). Briefly, roots of the CK44 transgenic and wild-type lines were harvested 48 h after transplanting and placed in a solution containing 1% high- and 1% low-gelling temperature agarose (1:1) in 1 mM CaSO_4_, which solidified at approximately 30°C, and 30 μM H_2_DCF. As the solution cooled, roots were immersed immediately before the onset of solidification. To avoid osmotic shock, for the water-stressed roots the solution water potential was lowered to −1.6 MPa (the water potential of the vermiculite in which the roots had been growing) using melibiose. Melibiose was used for this purpose because of evidence that it is neither hydrolyzed nor taken up by plant cells (Dracup et al., 1986). After 30 min, agarose blocks containing the root apical 20 mm were removed and H_2_DCF fluorescence in epidermal cells was imaged at 1.5–2.0 mm from the apex using two-photon laser-scanning confocal microscopy (Zeiss LSM NLO 510 combined with a Coherent, Chameleon 720–950 nm laser). Seven optical sections (5 μm in thickness) were merged into a single image for each root, and normalized fluorescence intensity (sum of pixel intensities in a defined area divided by pixel number) was quantified using the threshold option of Metamorph software (Molecular Devices Inc.).

### Cytosolic ROS

Cytosolic ROS was imaged by staining with the membrane-permeable fluorescent dye carboxy-H_2_DCFDA (Molecular Probes). Briefly, roots were harvested 48 h after transplanting, placed in a solution containing 1 mM CaSO_4_ and 15 μM carboxy-H_2_DCFDA, and stained for 30 min. For the water-stressed roots, melibiose was added to lower the solution water potential to −1.6 MPa. The apical 10 mm were then imaged for carboxy-H_2_DCFDA fluorescence using a stereomicroscope (Leica MZFLIII) with a GFP filter (excitation 488 nm, emission 515/30 nm). Normalized fluorescence intensity was quantified as described above.

### Cell Length and Relative Elongation Rate Profiles

Spatial distributions of relative elongation rate (h^–1^) were calculated from root elongation rates and cell length profiles (Silk et al., 1989; Yamaguchi et al., 2010). Accurate determination of relative elongation rate profiles from anatomical records requires steady growth and cell length distribution. Root elongation was essentially steady in well-watered and water-stressed roots after 12 and 24 h from transplanting, respectively. However, because the water-stressed roots elongated more slowly, the harvest time was increased from 48 h in well-watered roots to 72 h in water-stressed roots to allow greater time for stabilization of the cell length profile.

Briefly, 25–30 seedlings were transplanted to well-watered or water-stressed conditions, and root elongation rates were measured from 36–48 h to 48–72 h after transplanting, respectively. In each treatment, several roots that were straight and had an elongation rate similar to the mean were selected, and the apical 15 mm were sectioned longitudinally (125 μm in thickness) using a Vibratome (Vibratome 3000 plus), stained with 1 mg mL^–1^ Calcofluor (Sigma-Aldrich) for 15 min to visualize the cell walls, and imaged by confocal microscopy (Yamaguchi et al., 2010). The spatial distribution of cortical cell length for each root was determined from 4 to 12 cells per position (well-watered: 0.5-mm intervals to 4 mm from the apex, then at 1-mm intervals; water-stressed: 0.25-mm intervals to 4 mm from the apex, then at 1-mm intervals) until unchanging mean lengths were obtained.

Spatial distributions of displacement velocity (mm h^–1^) were calculated using the relationship *L*_A_/*L*_F_ = *V*_A_/*V*_F_, where *L*_A_ is mean cell length at position A, *L*_F_ is final cell length (average of the 4–6 most distal measurement positions), *V*_A_ is velocity at position A, and *V*_F_ is final velocity (equal to the root elongation rate) (Silk et al., 1989). This method cannot accurately calculate displacement velocities in the meristem because cell lengths in this region are determined by both elongation and division. Therefore, displacement velocities were calculated from the distal end of the meristem, approximated as the position where cell lengths reached 2.5 times the length of the shortest cells (Erickson, 1961). Velocity profiles were obtained for individual roots, and sigmoidal (Slogistic 1) curves were fitted using Origin software (OriginLab Corp.) using the function:

$$y=a/\left[1+{\text{e}}^{-K\,\left(x-x\,c\right)}\right]$$

where *x* is distance from the root cap junction and *a*, *K*, and *xc* are parameters obtained from the non-linear fit of the curve. The goodness of fit (*R*^2^) for each velocity profile was >0.98. Derivatives of the curves provided relative elongation rate profiles for each root, which were averaged to obtain mean profiles per treatment.

Residence times for cells within the growth zone (beyond the meristem) were obtained according to Dumlao et al. (2015). Briefly, using the analytical function described above, interpolated displacement velocities were calculated at 0.25 mm intervals on an individual root basis. Local displacement times were calculated from the interpolated velocities (local displacement time = 0.25/local velocity) and were then numerically integrated to obtain growth trajectories (position versus time). The relative elongation rate associated with each position was then plotted against time to provide the temporal pattern of relative elongation rate as cells were displaced from the distal end of the meristem to the end of the growth zone.

### Cell Production Rate

Rates of cell flux (cells h^–1^) were calculated by dividing root elongation rates by final cell lengths; under steady conditions, cell flux approximates the cell production rate (Silk et al., 1989). Since cell length profiles in the growth zone were not obtained for the B73 lines, the end of the growth zone was not precisely determined for these roots. Therefore, to ensure that cell lengths were measured in regions where elongation had ceased, final cortical cell lengths were calculated by averaging mean cell lengths from 16 to 24 mm and 11 to 18 mm from the apex in well-watered and water-stressed roots, respectively.

### H_2_O_2_ Scavenger Experiments

Hydrogen peroxide scavenger experiments were conducted using maize inbred line FR697 due to the large number of seeds required combined with limited availability of seeds of the transgenic and wild-type lines. FR697 was previously used to characterize spatial patterns of transcript (Spollen et al., 2008), cell wall protein (Zhu et al., 2007), and apoplastic H_2_O_2_ (Voothuluru and Sharp, 2013) in well-watered and water-stressed primary roots. Seeds were germinated as described above, and seedlings with primary roots 5–20 mm in length were pretreated with the H_2_O_2_ scavenger potassium iodide (KI) (Liszkay et al., 2004; Encina and Fry, 2005). Plexiglas holders (32 cm long, 4 cm wide, three sides of 2 cm in height) were used to cast a 5-mm layer of 1% agarose gel containing 0, 30, or 45 mM KI, and the apical 3 mm of the roots were inserted into the edge of the gel (20 seedlings per holder) for 6 h. Because the roots elongated during the pretreatment, kernels were moved every 2 h such that only the apical 3 mm of the roots remained in the gel. Seedlings were then transplanted into vermiculite at water potentials of −0.03 or −1.6 MPa. Environmental conditions during pretreatment and after transplanting were as described above. In preliminary experiments, the effectiveness of several KI concentrations was examined for effects on root elongation after transplanting to the well-watered and water-stressed treatments. The 30 mM (as also used by Liszkay et al. (2004) in studies of the maize primary root growth zone) and 45 mM pretreatments were selected as being the most effective.

In each treatment, several roots that were straight and had an elongation rate similar to the mean were harvested 36 h after transplanting and used to measure final cell lengths and cell production rates, as described above. The growth zone length in FR697 is approximately 12 and 7 mm under well-watered and water-stressed (−1.6 MPa) conditions, respectively (Sharp et al., 2004). Accordingly, final cell lengths were calculated by averaging mean lengths at 1-mm intervals from 12 to 17 mm from the apex in well-watered roots, and at 2-mm intervals from 9 to 13 mm from the apex in water-stressed roots.

## Results

Unless otherwise noted, water stress was imposed by transplanting seedlings into vermiculite at a water potential of −1.6 MPa, as used in previous studies of ROS metabolism in water-stressed maize primary roots (Zhu et al., 2007; Voothuluru and Sharp, 2013).

### Spatial Profiles of Oxalate Oxidase Activity in *Oxalate Oxidase* Transgenic and Wild-Type Roots Under Well-Watered and Water-Stressed Conditions

**FIGURE 1:**
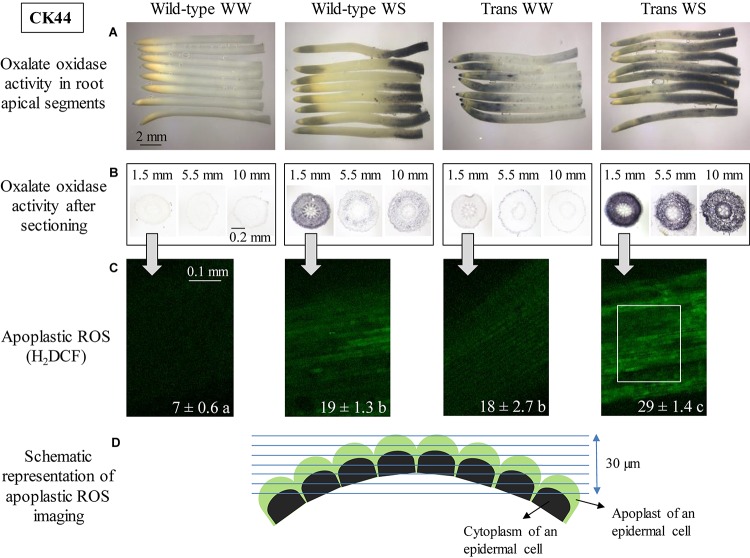
Oxalate oxidase activity and apoplastic ROS in the apical region of the primary root in CK44 *oxalate oxidase* transgenic (Trans) and wild-type lines at 48 h after transplanting to well-watered (WW) or water-stressed (WS; –1.6 MPa) conditions. Oxalate oxidase activity was measured by oxidation of 4-chloro-1-naphthol, seen as dark blue staining, in approximately 12-mm apical segments **(A)** and in 14-μm transverse sections at various distances from the root apex **(B)**. Apoplastic ROS **(C)** was stained using the fluorescent ROS indicator H_2_DCF (membrane impermeable); confocal images of the epidermis at 1.5–2.0 mm from the root apex are shown. The images are composed of projections of seven consecutive optical sections (5 μm in thickness) obtained by two-photon confocal microscopy. The numbers on the images indicate normalized pixel intensities (± SE; *n* = 3 roots) of a 0.04 mm^2^ area of the root (0.18 mm length, 0.23 mm width), as illustrated in the Trans WS image. Different letters indicate significant differences (*t*-test; *P* < 0.05). All experiments were repeated with similar results, and representative images are shown. Panel **D** shows a schematic representation of apoplastic ROS imaging in the epidermal cell file **(D)**. Green and black semicircles indicate the apoplast and cytoplasm of the epidermal cells, respectively, and blue lines indicate the consecutive optical planes at which fluorescence was captured. The schematic also illustrates the curvature of the root, which necessitated capture of fluorescence in multiple optical planes.

The spatial distribution of oxalate oxidase activity was determined in apical 12-mm segments of the primary root of CK44 wild-type and transgenic lines at 48 h after transplanting to well-watered or water-stressed conditions (Figure 1A). In all cases, this segment encompassed the growth zone, which was approximately 9 and 11 mm in length, respectively, in wild-type and transgenic roots in the well-watered treatment, and was shortened to 5 and 4 mm in wild-type and transgenic roots under water-stress (growth zone lengths are presented below). Consistent with previous results in a different genotype (inbred line FR697; Voothuluru and Sharp, 2013), the CK44 wild-type line showed no discernable staining for oxalate oxidase activity at any location in well-watered roots, whereas water-stressed roots exhibited a pronounced increase in activity in the apical 2-mm region immediately behind the root cap junction (Figure 1A). The water-stressed wild-type roots also showed an increase in oxalate oxidase activity from approximately 9 to 12 mm from the root apex, which was beyond the growth zone. Roots stained in buffer without oxalic acid showed no staining in either treatment, demonstrating that the staining was specific to oxalate oxidase activity (data not shown). The lack of staining in the no-oxalic acid control for the water-stressed roots was probably attributable to limited sensitivity of the assay, rather than lack of endogenous apoplastic oxalate.

In contrast to the lack of staining for oxalate oxidase activity in well-watered wild-type roots, well-watered roots of the CK44 transgenic line exhibited substantial activity in the apical region, extending to approximately 4 mm from the apex (Figure 1A). Faint and stippled staining also occurred throughout the rest of the segment. In the water-stressed treatment, the transgenic roots showed extended apical and basal regions of staining compared to the wild-type roots, such that staining occurred throughout most of the segment (Figure 1A). In both the wild-type and transgenic lines, staining patterns obtained at 72 h after transplanting to well-watered and water-stressed conditions were similar to those obtained at 48 h (Supplementary Figure S1), indicating that the region-specific patterns of staining were steady rather than transient events.

**FIGURE 2:**
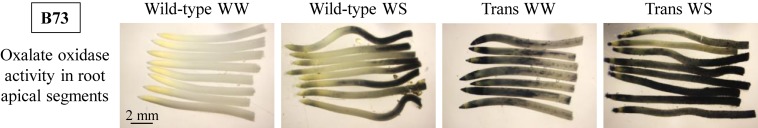
Oxalate oxidase activity measured by oxidation of 4-chloro-1-naphthol, seen as dark blue staining, in approximately 12-mm apical segments of the primary root in the B73 *oxalate oxidase* transgenic (Trans) and wild-type lines at 48 h after transplanting to well-watered (WW) or water-stressed (WS; –1.6 MPa) conditions. The experiments were repeated with similar results and representative images are shown.

Spatial patterns of staining for oxalate oxidase activity in the apical segments of the B73 wild-type and transgenic lines under well-watered and water-stressed conditions were similar to those observed in the CK44 lines (Figure 2). Well-watered wild-type roots did not exhibit staining at any location, whereas water-stressed wild-type roots showed increased activity in the apical 2-mm region, as well as in the basal region (generally beyond 8 mm from the apex, although with considerable variation in intensity and extent between individual roots). The transgenic line showed increased oxalate oxidase activity in the apical 6-mm region of well-watered roots, and throughout the segment in water-stressed roots.

To ensure that the spatial patterns of staining for oxalate oxidase activity observed in the intact root segments were not attributable to differential penetration of the staining solution into the different regions, transverse sections were made at several distances from the apex of well-watered and water-stressed roots of the CK44 wild-type and transgenic lines, and were stained after sectioning. The results were generally consistent with the staining patterns in the intact segments (Figure 1B). In well-watered wild-type roots, sections taken at 1.5, 5.5, and 10 mm from the apex showed minimal oxalate oxidase activity in all tissues, whereas in the water-stressed treatment, the wild-type line showed a substantial increase in activity at 1.5 mm from the apex, and mild and moderate staining, respectively, at 5.5 and 10 mm. It should be noted that at 10 mm from the apex of water-stressed roots, oxalate oxidase activity was transitioning from the region of minimal activity to the region of stronger activity at the basal end of the segments (Figure 1A), and thus corresponded to the moderate intensity of staining observed in the transverse section at this location. In all locations, staining was more pronounced in the cortex and epidermis than in the stele. In the transgenic line, well-watered roots showed moderate staining in all tissues at 1.5 mm from the apex, and low levels of staining at the other locations. Water-stressed roots of the transgenic line exhibited a pronounced increase in staining in the cortex and epidermis at 1.5 mm from the apex, and also showed substantial staining at both 5.5 and 10 mm.

To assess the relationship of the spatial profiles of oxalate oxidase activity with transgene expression, qRT-PCR experiments were conducted with the CK44 transgenic line at 36 and 48 h after transplanting to well-watered and water-stressed conditions. In well-watered roots, transgene expression was about threefold higher in the apical 0–5 mm region compared with the 5–12 mm region (Table 1). In water-stressed roots, similarly, transgene expression was fourfold higher in the 0–5 mm region compared with the 5–12 mm region. Accordingly, the increased oxalate oxidase activity in the apical compared with the basal region of both well-watered and water-stressed transgenic roots could be explained, at least partly, by the greater *oxalate oxidase* transgene expression in the apical region. Interestingly, the results also showed that in both the apical and basal regions of the water-stressed roots, *oxalate oxidase* transgene transcript abundance was 30–50% lower than in well-watered roots.

**TABLE 1:** Transgene expression quantification by qRT-PCR.

**Treatment**	**Distance from apex**	**2^–[Δ][Δ]Ct^ values for transgene expression**	
		**36 h**	**48 h**
Well-watered	0–5 mm	0.73 ± 0.06**	0.64 ± 0.07*
	5–12 mm	0.21 ± 0.02	0.20 ± 0.02
Water-stressed	0–5 mm	0.43 ± 0.01^*▲▲^	0.45 ± 0.07*
	5–12 mm	0.10 ± 0.04^▲^	0.12 ± 0.01^▲▲^

2^–[Δ][Δ]*Ct*^ values in the apical (0–5 mm from the apex) and basal (5–12 mm from the apex) regions of the primary root in the CK44 *oxalate oxidase* transgenic line at 36 or 48 h after transplanting to well-watered or water-stressed (−1.6 MPa) conditions. Data are means ± SE of three samples. Asterisks denote significant differences between the 0–5 mm and 5–12 mm regions (*t*-test; * *P* < 0.05; ** *P* < 0.01). Triangles denote significant differences between well-watered and water-stressed treatments (*t*-test; ^▲^*P* < 0.1; ^▲▲^*P* < 0.05).

### Apoplastic ROS Levels in *Oxalate Oxidase* Transgenic and Wild-Type Roots Under Well-Watered and Water-Stressed Conditions

It was previously shown that the increase in oxalate oxidase activity in the apical region of the growth zone in water-stressed maize primary roots was associated with a pronounced increase in the level of apoplastic H_2_O_2_ compared with the well-watered control (Zhu et al., 2007; Voothuluru and Sharp, 2013). Accordingly, the increases in oxalate oxidase activity observed in the apical region of both well-watered and water-stressed roots of the CK44 transgenic line predicted an increase in apoplastic H_2_O_2_ levels under both conditions. This hypothesis was tested by *in situ* confocal imaging of apoplastic ROS in epidermal cells of the apical region (1.5–2 mm from the apex) using the membrane-impermeable fluorescent indicator dye H_2_DCF. Consistent with previous results using this technique (in line FR697; Zhu et al., 2007), the level of apoplastic ROS in water-stressed roots of the wild-type was higher (approximately threefold) than in well-watered roots (Figure 1C). Moreover, as anticipated, both well-watered and water-stressed roots of the transgenic line exhibited substantially increased apoplastic ROS levels compared to the wild-type controls. Evidence of apoplastic localization of H_2_DCF staining was provided by analysis of the pattern of staining in consecutive focal planes, which showed no evidence of intracellular staining (Figure 1D and Supplementary Video S1). The variation in apoplastic ROS levels among the different lines and treatments correlated closely with the relative intensity of staining for oxalate oxidase activity (especially with that in the transverse sections; Figure 1B). Since the oxalate oxidase protein is specifically involved in production of apoplastic H_2_O_2_ (Davidson et al., 2009), the observed increases in fluorescence in the apical region of the transgenic line under both well-watered and water-stressed conditions likely reflect increases in apoplastic H_2_O_2_, although the possible involvement of other ROS cannot be excluded. Levels of apoplastic ROS were not assessed in the basal region of the growth zone because of evidence of membrane permeability to H_2_DCF in the epidermal cells of this region (Zhu et al., 2007).

To assess whether the CK44 transgenic line also exhibited increases in cytosolic ROS levels, well-watered and water-stressed roots were stained with the membrane-permeable dye carboxy-H_2_DCFDA. Cytosolic ROS levels were not increased in roots of the transgenic compared to the wild-type line in either the well-watered or the water-stressed condition (Supplementary Figure S2). Although there was a marginal increase in cytosolic ROS levels in the apical 2.5 mm region of water-stressed compared with well-watered roots in both the transgenic and wild-type lines, the increases were not significant.

### Root Elongation in *Oxalate Oxidase* Transgenic Lines Responds Differentially to Well-Watered or Water-Stressed Conditions

**FIGURE 3:**
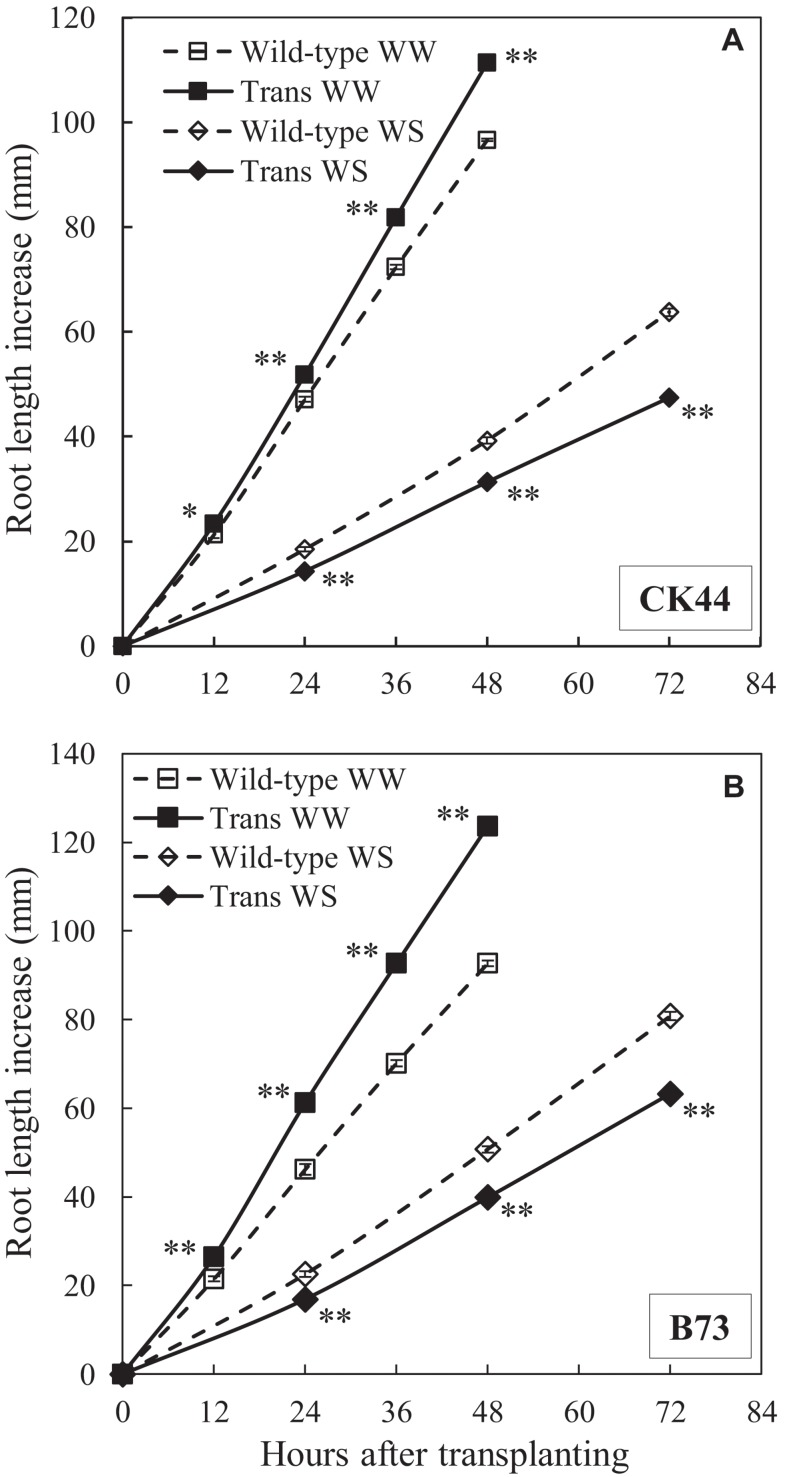
Primary root elongation of CK44 **(A)** and B73 **(B)** *oxalate oxidase* transgenic (Trans) and wild-type lines after transplanting to well-watered (WW) or water-stressed (WS; –1.6 MPa) conditions. Data are means ± SE (*n* = 10–32). Asterisks denote significant differences between the transgenic and wild-type lines (*t*-test; ^∗^*P* < 0.05; ^∗∗^*P* < 0.01). The experiment was repeated three (CK44) or two (B73) times with similar results.

Under well-watered conditions, primary root elongation in the CK44 transgenic line was increased compared with the wild-type throughout the experiments (Figure 3A). At 48 h after transplanting, the root length of the transgenic was 13% greater than that of the wild-type (111 ± 2 mm versus 98 ± 1 mm; data are means ± SE of four experiments). The increase in root elongation was accompanied by a 17% increase in shoot elongation (shoot lengths were 125 ± 2 mm in the transgenic compared with 107 ± 3 mm in the wild-type). In the water stress treatment, in contrast, root elongation of the transgenic line was consistently reduced compared with the wild-type, such that root length at 72 h after transplanting was 24% less in the transgenic than in the wild-type (44 ± 2 mm versus 58 ± 3 mm; data are means ± SE of three experiments). Shoot growth was completely inhibited in both the wild-type and transgenic lines due to the severity of the water stress treatment (Sharp et al., 1988).

Similar results were obtained with the B73 transgenic and wild-type lines (Figure 3B). The transgenic showed a 33% increase in root length at 48 h in the well-watered treatment and a 22% decrease in root length at 72 h in the water stress treatment compared with the wild-type. Root elongation rates were approximately steady after 12 and 24 h from transplanting to the well-watered and water-stressed conditions, respectively, in both the CK44 and B73 transgenic and wild-type lines (Supplementary Tables S1, S2).

**FIGURE 4:**
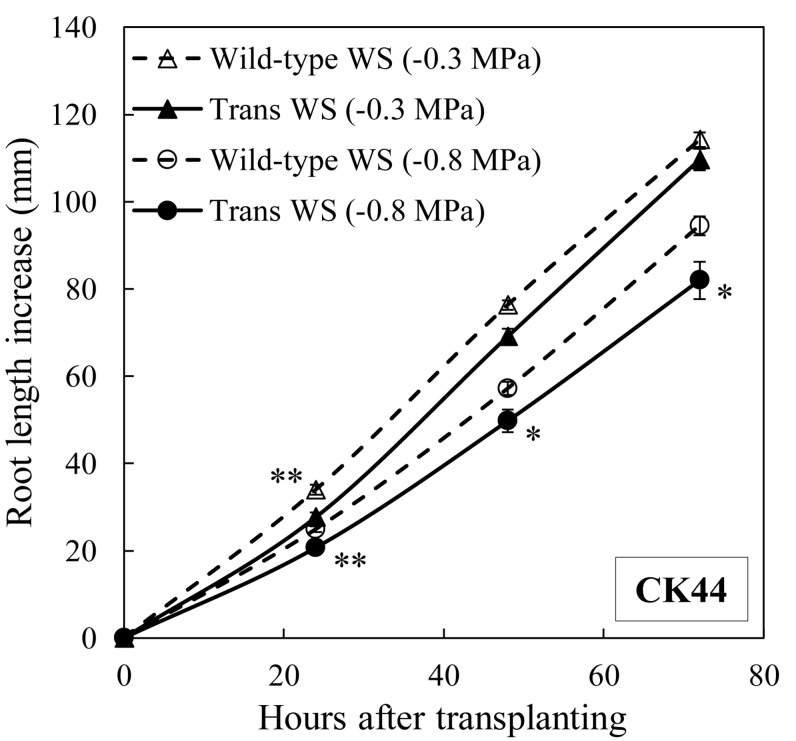
Primary root elongation of CK44 *oxalate oxidase* transgenic and wild-type lines after transplanting to mild (–0.3 MPa) or moderate (–0.8 MPa) water stress conditions. Data are means ± SE (*n* = 14–20). Asterisks denote significant differences between the transgenic and wild-type lines (*t*-test; ^∗^*P* < 0.05; ^∗∗^*P* < 0.01).

The responses of primary root elongation were also evaluated under mild (water potential of −0.3 MPa) and moderate (water potential of −0.8 MPa) water stress levels in the CK44 transgenic and wild-type lines (Figure 4). In the mild stress condition, root length was slightly reduced in the transgenic compared with the wild-type line throughout the experiment, although this effect was significant only at the 24 h time point. In the moderate stress treatment, root length was consistently reduced (by 13% at 72 h after transplanting) in the transgenic compared with the wild-type. Taking into account the fact that root elongation in the transgenic line was promoted in the well-watered condition, these results show that inhibition of root elongation under water stress was considerably greater in the transgenic compared with the wild-type line. At 48 h after transplanting, the transgenic line exhibited decreases in root length of 38, 55, and 72% under mild, moderate and severe stress conditions, respectively. In the wild-type, in contrast, root length was inhibited by only 21, 41, and 59%, respectively, in these treatments. Because the transgenic line showed decreased root elongation at all of the water stress levels tested, further experiments were conducted only under the severe water stress (−1.6 MPa) condition.

Taken together, the results shown in Figures 1–4 indicate that the oxalate oxidase-mediated increase in apoplastic H_2_O_2_ in the growth zone of the transgenic lines positively and negatively modulated root growth under well-watered and water-stressed conditions, respectively.

### Kinematic Analysis of *Oxalate Oxidase* Transgenic and Wild-Type Roots Under Well-Watered and Water-Stressed Conditions

The overall rate of root elongation is determined by the product of cell flux (the rate per cell file at which cells exit the growth zone) and final cell length. Under steady conditions, the cell flux approximates the rate of cell production (the rate per cell file at which cells leave the meristem; Silk et al., 1989). Accordingly, cortical cell length profiles were obtained for the CK44 transgenic and wild-type lines to determine whether the differential effects of enhanced oxalate oxidase activity/increased apoplastic H_2_O_2_ on root elongation under well-watered and water-stressed conditions were primarily attributable to changes in final cell length and/or cell production, and also to localize any effects on the spatial distribution of relative elongation rate within the growth zone (Sharp et al.1988; Silk et al., 1989).

**FIGURE 5:**
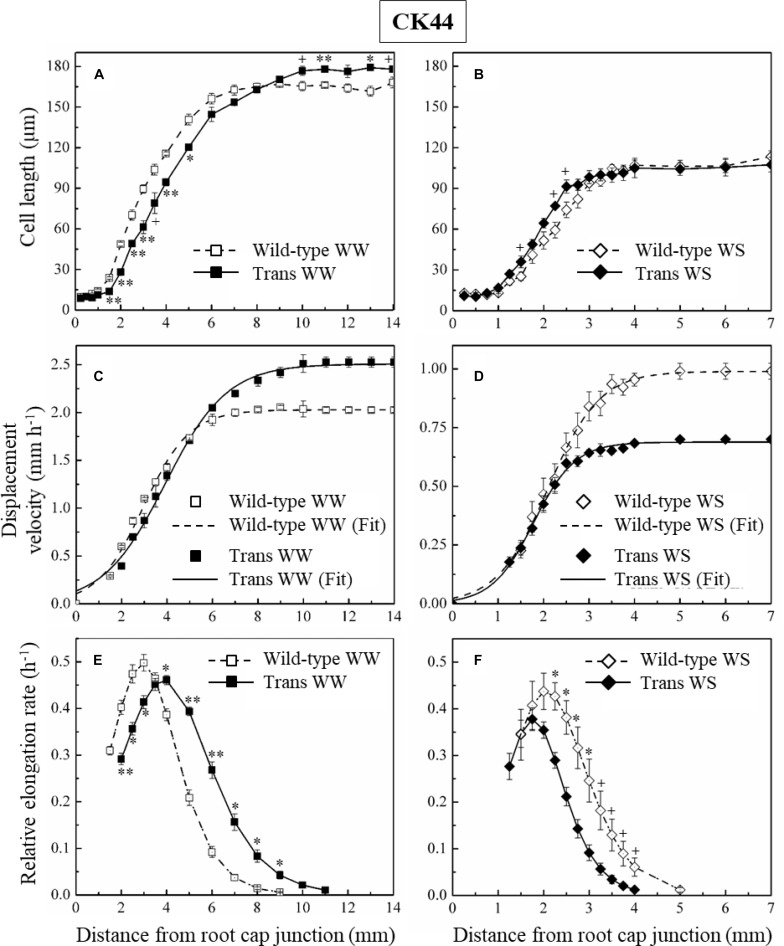
Cortical cell length profiles **(A,B)**, displacement velocity profiles **(C,D)**, and relative elongation rate profiles **(E,F)** as a function of distance from the root cap junction in the primary root of CK44 *oxalate oxidase* transgenic (Trans) and wild-type lines growing under well-watered (WW) or water-stressed (WS; –1.6 MPa) conditions. (In the water-stressed roots, cell lengths were also measured from 8 to 10 mm from the apex, but are not shown for clarity). Derivatives of sigmoidal curves fitted to the displacement velocity profiles of individual roots were used to obtain the mean relative elongation rate profiles. For clarity, only the curves fitting the mean velocity profiles (Fit) are shown, for which the goodness of fit (*R*^2^) was >0.99 in all cases. Roots were harvested 48 h after transplanting to well-watered conditions and 72 h after transplanting to water-stressed conditions; values are means ± SE of three to four roots. Significant differences between the transgenic and wild-type lines are shown for the cell length and relative elongation rate data (*t*-test; ^+^*P* < 0.1; ^∗^*P* < 0.05; ^∗∗^*P* < 0.01). The experiments were repeated with similar results.

In the well-watered treatment, despite the fact that the transgenic line exhibited a 25% increase in root elongation rate compared with the wild-type during the period of steady growth before harvest, the final cell length at the end of the growth zone was only 5% greater in the transgenic (Figure 5A and Table 2). In contrast, the estimated cell production rate was 19% greater in the transgenic line (Table 2). The cell length profile also revealed a basal shift in the location at which final cell length was achieved in the transgenic compared with the wild-type (Figure 5A), such that the length of the growth zone increased by 22% from 9 mm in the wild-type to 11 mm in the transgenic line, approximately reflecting the increase in root elongation rate. The lengthening of the growth zone was also reflected in the profiles of displacement velocity (Figure 5C) and relative elongation rate (Figure 5E), which showed that local relative elongation rates were lower in the apical region and higher in the basal region of the transgenic compared to the wild-type. In contrast, the maximum relative elongation rate was not significantly different between the lines (Figure 5E).

**TABLE 2:** Elongation rate, final cell length, and cell production rate of the primary root in the CK44 *oxalate oxidase* transgenic and wild-type lines at 48 or 72 h after transplanting to well-watered or water-stressed (−1.6 MPa) conditions, respectively.

	**CK44, well-watered**			**CK44, water-stressed**		
	**Wild-type**	**Transgenic**	**Change compared to wild-type (%)**	**Wild-type**	**Transgenic**	**Change compared to wild-type (%)**
Root elongation rate (mm h^–1^)	2.03 ± 0.03	2.53 ± 0.05**	25	0.99 ± 0.02	0.70 ± 0.01**	−29
Final cell length (μm)	165 ± 2	173 ± 1*	5	111 ± 5	107 ± 6	−4
Cell production rate (cells h^–1^)	12.3 ± 0.2	14.6 ± 0.3**	19	8.9 ± 0.2	6.6 ± 0.4**	−26

Elongation rates were calculated for the preceding 12 h (well-watered) or 24 h (water-stressed) periods. Values are means ± SE of three to four roots. Asterisks denote significant differences between the transgenic and wild-type lines (*t*-test; * *P* < 0.05; ** *P* < 0.01).

In the water-stressed treatment, the root elongation rate was decreased by 29% in the transgenic compared with the wild-type, but final cell length was not significantly different between the lines (Figure 5B and Table 2). In contrast, the estimated cell production rate was decreased by 26% in the transgenic compared with the wild-type line (Table 2). Consistent with previous studies (Sharp et al., 1988; Liang et al., 1997), the growth zone was substantially shorter in the water-stressed compared with well-watered roots in both the transgenic and wild-type lines (Figures 5A,B). Opposite to the effect in well-watered transgenic roots, however, the profiles of cell length, displacement velocity and relative elongation rate were shifted apically in the transgenic line under water stress, reflecting a further shortening of the growth zone from 5 to 4 mm (Figures 5B,D,F). The peak relative elongation rate was slightly decreased in the transgenic compared with the wild-type, although this difference was not significant (*P* = 0.17).

In the B73 transgenic line, effects on cell length and cell production rate under well-watered and water-stressed conditions were consistent with those observed in the CK44 background. In this case, only final cell lengths (Supplementary Figure S3) rather than complete cell length profiles were measured. In well-watered roots, the cell production rate in the transgenic was 22% higher than in the wild-type (Table 3). In addition, the transgenic line showed a 13% increase in final cell length under the well-watered condition. In the water-stressed roots, in contrast, cell production rate was 17% lower in the transgenic compared with the wild-type (Table 3). Final cell lengths were slightly but not significantly (*P* = 0.12) decreased in the transgenic line in the water-stressed condition.

**TABLE 3:** Elongation rate, final cell length, and cell production rate of the primary root in the B73 *oxalate oxidase* transgenic and wild-type lines at 48 or 72 h after transplanting to well-watered or water-stressed (−1.6 MPa) conditions, respectively.

	**B73, well-watered**			**B73, water-stressed**		
	**Wild-type**	**Transgenic**	**Change compared to wild-type (%)**	**Wild-type**	**Transgenic**	**Change compared to wild-type (%)**
Root elongation rate (mm h^–1^)	1.86 ± 0.03	2.56 ± 0.04**	38	1.24 ± 0.01	0.96 ± 0.06**	−23
Final cell length (μm)	187 ± 1	210 ± 2**	13	142 ± 2	132 ± 4	−7
Cell production rate (cells h^–1^)	10.0 ± 0.1	12.2 ± 0.2**	22	8.8 ± 0.2	7.2 ± 0.2*	−17

Elongation rates were calculated for the preceding 12 h (well-watered) or 24 h (water-stressed) periods. Values are means ± SE of three to four roots. Asterisks denote significant differences between the transgenic and wild-type lines (*t*-test; * *P* < 0.05; ** *P* < 0.01).

Taking into account that rates of cell production were increased in the well-watered roots, the analysis revealed that cell production was more sensitive to water stress in the transgenic lines compared with the wild-type lines. In the CK44 background, the transgenic showed a 55% decrease in cell production rate in water-stressed compared with well-watered roots, while the wild-type showed only a 28% decrease (Table 2). Similarly, in the B73 background, cell production rate was decreased by 41% in the transgenic but by only 12% in the wild-type (Table 3). In contrast, the difference in the response of final cell length to water-stress was less pronounced between the transgenic and wild-type lines, being inhibited by 38 and 33% in the CK44 background and by 37 and 24% in the B73 background, respectively.

**FIGURE 6:**
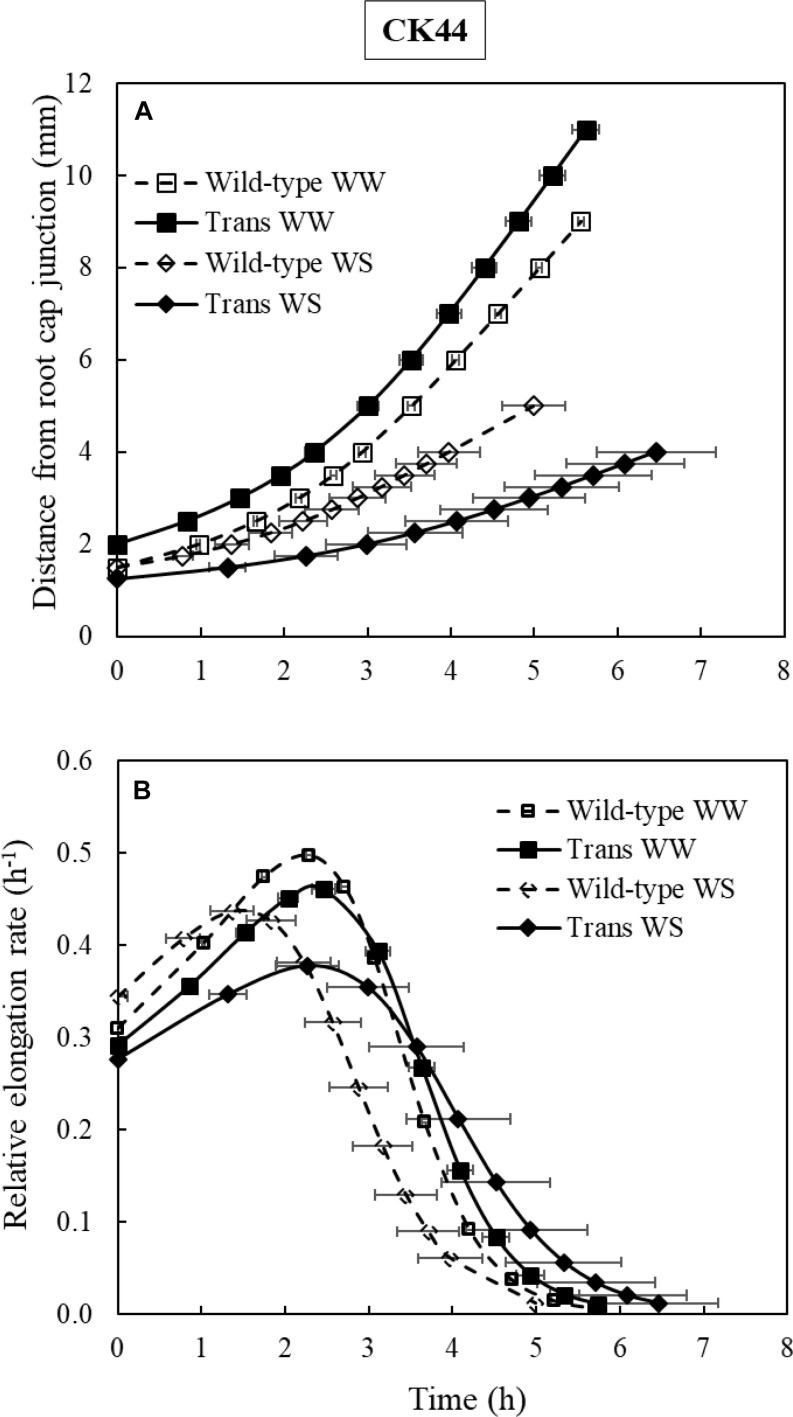
Growth trajectories **(A)** and temporal relative elongation rate profiles **(B)** of the primary root of CK44 *oxalate oxidase* transgenic (Trans) and wild-type lines growing under well-watered (WW) or water-stressed (WS; –1.6 MPa) conditions. Growth trajectories were used to determine the times required for cells to move from the distal end of the meristem to more basal locations, and were obtained by plotting position versus the integrated local displacement time. The relative elongation rate corresponding to a particular position (Figures 5E,F) was then plotted against the integrated displacement time to obtain the temporal relative elongation rate profiles **(B)**. The total residence times of cells within the growth zone were not significantly different between the transgenic and wild-type lines in either well-watered or water-stressed comparisons (*t*-test; *P* > 0.1).

To analyze whether oxalate oxidase-mediated increases in apoplastic H_2_O_2_ impacted temporal aspects of cell elongation, time courses of relative elongation rate in the root growth zone of CK44 transgenic and wild-type lines growing under well-watered or water-stressed conditions were calculated (Figure 6). The results show that the temporal patterns of relative elongation rate were similar between the lines in both treatments, and moreover, that the total residence time for cells to move from the distal end of the meristem to the end of the growth zone was not significantly different (*P* > 0.2) between the lines and treatments. Thus, cessation of elongation occurred in tissue of approximately the same age regardless of the modifications of growth zone length that occurred in the different treatments.

### Apoplastic H_2_O_2_ Removal Differentially Affects Cell Production and Root Elongation Under Well-Watered and Water-Stressed Conditions

As detailed above, the increases in apoplastic H_2_O_2_ in the root growth zone in the transgenic lines resulted in differential effects on cell production and root elongation in well-watered (both processes increased) and water-stressed (both processes decreased) roots. Accordingly, these findings suggest that the normal increase in apoplastic H_2_O_2_ in water-stressed roots may be involved in down-regulation of cell production and root elongation. On the other hand, it is also possible that the additional increase in apoplastic H_2_O_2_ that occurred in the transgenic lines under water-stress (Figure 1C) may have caused excess H_2_O_2_ levels, leading to a spurious inhibition of cell production and root elongation and obscuring the function of the normal increase in H_2_O_2_. To distinguish between these alternate possibilities, an additional set of experiments was conducted to examine the effects of removing apoplastic H_2_O_2_ from the apical region of the growth zone in non-transgenic roots growing under well-watered and water-stressed conditions. Based on the transgenic results, it was hypothesized that H_2_O_2_ removal would lead to decreased rates of cell production and root elongation under well-watered conditions, and conversely, to increased rates of cell production and root elongation under water stress. The results, as follows, were consistent with this hypothesis.

**FIGURE 7:**
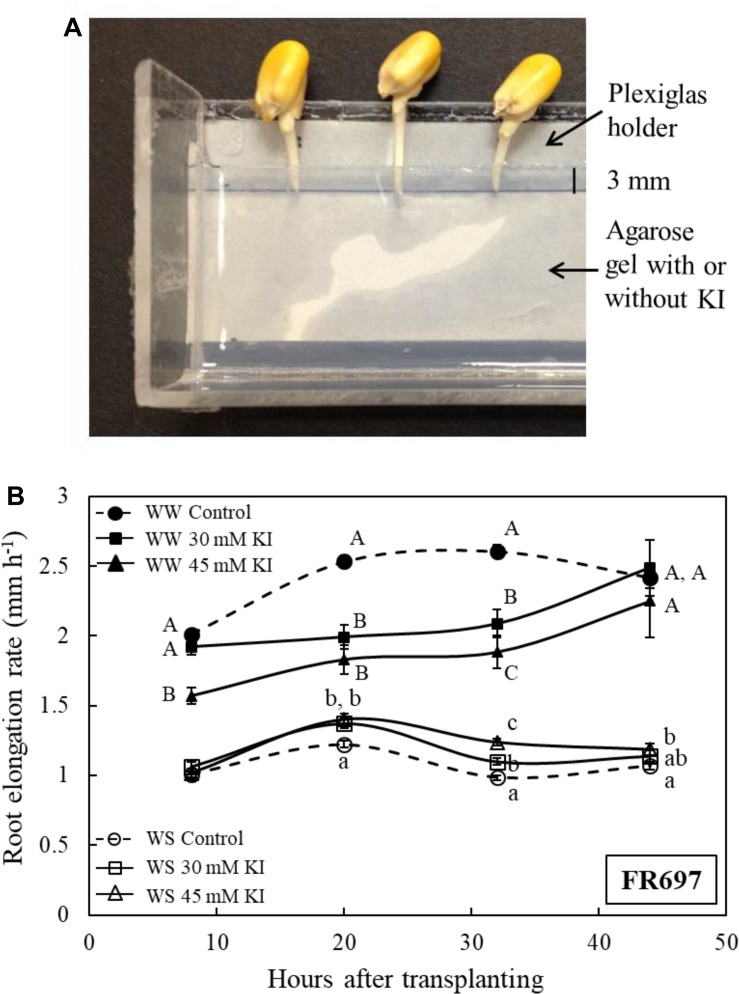
**(A)** Part of the Plexiglas holder, viewed from above, which was used to pretreat the apical 0–3 mm region of the primary root of line FR697 with the H_2_O_2_ scavenger KI. The holder contained a 5-mm thick layer of 1% agarose gel, with or without KI, into which the root tip was inserted. Because the roots elongated during the pretreatment, the kernels were moved away from the gel every 2 h such that only the apical 3 mm of the roots were embedded in the gel. After 6 h, the seedlings were transplanted to well-watered or water-stressed conditions. **(B)** Elongation rates of control and KI-pretreated (30 or 45 mM) primary roots after transplanting to well-watered (WW) or water-stressed (WS; –1.6 MPa) conditions. The elongation rates are plotted at the midpoint between marking intervals, which were at 0, 16, 24, 40, and 48 h after transplanting. Data are means ± SE of three independent experiments (*n* = 15–40 seedlings per treatment in each experiment). Different letters denote significant differences between treatments at specific times within each condition (*t*-test; *P* < 0.05).

These experiments were conducted using maize inbred line FR697, which was shown previously to exhibit a pronounced increase in apoplastic H_2_O_2_ in the apical region of the root growth zone under water-stressed conditions (Voothuluru and Sharp, 2013). To decrease H_2_O_2_ levels, the roots were pretreated with KI, which has been used in previous studies to scavenge H_2_O_2_ from cell walls (Nose et al., 1995; Encina and Fry, 2005; Dunand et al., 2007), including from the growth zone of the maize primary root (Liszkay et al., 2004). In order to scavenge H_2_O_2_ specifically in the apical region of the growth zone, the roots were pretreated by placing the apical 3 mm into agarose gel containing either 30 or 45 mM KI for 6 h (Figure 7A), and the seedlings were then transplanted as usual to well-watered or water-stressed conditions.

In both well-watered and water-stressed seedlings, effects of the KI pretreatment on root elongation were opposite to the effects of enhanced H_2_O_2_ that were observed in the transgenic lines. Thus, after transplanting to the well-watered condition, root elongation was substantially inhibited in KI-pretreated roots compared with the untreated control; from 24 to 40 h, elongation rates were decreased by 20 and 28%, respectively, following the 30 and 45 mM pretreatments (Figure 7B). Conversely, after transplanting to the water-stressed condition, KI-pretreated roots showed a significant increase in elongation compared with the control; from 24 to 40 h, elongation rates were increased by 12 and 26%, respectively, following the 30 and 45 mM pretreatments. In both the well-watered and water-stressed seedlings, effects of the KI pretreatment on root elongation diminished after 40 h from transplanting (Figure 7B).

To evaluate the relative effects of the scavenger pretreatment on final cell length and cell production rate, cortical cell length measurements were made 36 h after transplanting in roots pretreated with 0, 30 mM (well-watered), or 45 mM (water-stressed) KI (Supplementary Figure S4); these pretreatments resulted in almost steady root elongation during the preceding 20 h (Figure 7B). In well-watered roots, the KI pretreatment caused a 16% decrease in cell production rate whereas final cell length was not significantly decreased (Table 4). Conversely, in the water-stressed roots, the KI pretreatment resulted in a 20% increase in cell production rate, which, together with a 15% increase in final cell length, accounted for the 38% increase in root elongation observed in this experiment (Table 4).

**TABLE 4:** Elongation rate, final cell length, and cell production rate of control and KI-treated primary roots in line FR697 at 36 h after transplanting to well-watered or water-stressed (−1.6 MPa) conditions.

	**FR697, well-watered**			**FR697, water-stressed**		
	**Control**	**30 mM KI**	**Change compared to control (%)**	**Control**	**45 mM KI**	**Change compared to control (%)**
Root elongation rate (mm h^–1^)	2.57 ± 0.02	2.07 ± 0.05**	−20	1.00 ± 0.00	1.38 ± 0.00**	38
Final cell length (μm)	190 ± 3	182 ± 8	−4	142 ± 4	164 ± 6**	15
Cell production rate (cells h^–1^)	13.6 ± 0.3	11.5 ± 0.5**	−16	7.1 ± 0.2	8.5 ± 0.4*	20

Elongation rates were calculated for the preceding 14 h (well-watered) or 8 h (water-stressed) periods. Values are means ± SE of five to six roots. Asterisks denote significant differences between the control and KI-treated roots (*t*-test; * *P* < 0.05; ** *P* < 0.01). Roots were pretreated with 30 mM and 45 mM KI for well-watered and water-stressed roots, respectively, since these pretreatments resulted in almost steady root elongation during the preceding 20 h (Figure 7B).

## Discussion

### Spatial Variability of Oxalate Oxidase Activity in the Growth Zone of Wild-Type and *Oxalate Oxidase* Transgenic Roots Under Well-Watered and Water-Stressed Conditions

In this study, transgenic maize lines constitutively expressing a wheat *oxalate oxidase* (Ramputh et al., 2002; Mao et al., 2007), in combination with H_2_O_2_ scavenger experiments and kinematic growth analysis, were used to investigate the involvement of apoplastic H_2_O_2_ in regulating maize primary root elongation under well-watered and water-stressed conditions. The rationale for the use of the transgenic lines was to provide reliable increases in oxalate oxidase activity and apoplastic H_2_O_2_ in the root growth zone under both well-watered and water-stressed conditions. Transgene expression was under the control of the rice actin promoter, which is considered to have a constitutive expression pattern (McElroy et al., 1991). Nevertheless, marked spatial variability in the pattern of oxalate oxidase activity was observed in the transgenic as well as the wild-type roots.

In water-stressed wild-type roots (both the CK44 and B73 backgrounds), oxalate oxidase activity increased substantially only in the apical few mm of the growth zone (Figures 1A,B, 2), in agreement with previous findings in inbred line FR697 (Voothuluru and Sharp, 2013). This region encompassed the meristem, which was shown to extend approximately 2 mm from the apex in well-watered maize primary roots, and to be shortened under water stress (Sacks et al., 1997). The water-stressed roots also showed a pronounced increase in oxalate oxidase activity from approximately 9 to 12 mm from the root apex; however, this region was beyond the growth zone (Figure 5), and thus the increase in activity may be involved in cell wall maturation rather than growth regulatory processes (Caliskan and Cuming, 1998; Fry, 2004). Interestingly, the minimal activity in the basal region of the growth zone in water-stressed roots occurred despite increases in gene expression (Spollen et al., 2008) and protein abundance (Zhu et al., 2007) of oxalate oxidases. Further, the disparity in protein abundance and activity cannot be explained by spatially variable apoplastic pH (Fan and Neumann, 2004) or substrate availability, since the assay conditions controlled for these factors (Voothuluru and Sharp, 2013).

In well-watered transgenic roots, pronounced oxalate oxidase activity was observed only in the apical 4 mm (CK44) or 6 mm (B73) regions. Transgene expression was threefold greater in the apical compared to the basal region (Table 1), which could at least partly explain the activity profile. In the water-stressed transgenic roots, in contrast, there was a substantial increase in oxalate oxidase activity throughout most of the apical 12 mm, which appeared to reflect more than an additive effect of the normal increase in activity observed in wild-type roots plus the activity attributable to the transgene observed in well-watered transgenic roots (Figure 1A,B). Moreover, the increased activity occurred despite the fact that transgene expression was substantially reduced in both the apical and basal regions of water-stressed compared with well-watered roots (Table 1). The latter result suggests that the actin promoter may in fact respond to water deficit conditions; however, the factors underlying the spatial pattern and stress-responsiveness of transgene expression are beyond the scope of this study.

Collectively, these results suggest that the spatial patterns of oxalate oxidase activity in both well-watered and water-stressed transgenic roots, as well as in the water-stressed wild-type, likely involved unknown processes of post-translational regulation or metabolic control of enzyme activity. Despite this complexity, the apical region of the growth zone of both well-watered and water-stressed transgenic roots exhibited substantially increased apoplastic H_2_O_2_ compared with the respective wild-type roots (Figure 1C).

### Apoplastic H_2_O_2_ Modulates Root Elongation via Effects on Cell Production and Spatial Profiles of Cell Elongation

The oxalate oxidase transgenic lines showed an increase in root elongation under well-watered conditions and a decrease in root elongation under water-stressed conditions (Figures 3, 4). Kinematic analyses indicated that these differential effects on root elongation involved substantial effects on both cell production rate and the spatial profiles of cell elongation. Cell production rate was positively and negatively modulated under well-watered and water-stressed conditions, respectively, in close proportion to the increases and decreases in root elongation rate (Tables 2, 3). Conversely, scavenging of apoplastic H_2_O_2_ in the apical region of the growth zone by pretreatment with KI resulted in a decrease of elongation in well-watered roots and an increase of elongation in water-stressed roots (Figure 7B), and these responses were also accompanied by substantial effects on cell production rate (Table 4). Effects on overall cell elongation, as reflected in final cell lengths, were relatively minor in the transgenic lines and in the scavenger-treated roots under both well-watered and water-stressed conditions (Tables 2–4).

In contrast to the minor effects on overall cell elongation, however, spatial profiles of relative elongation rate were markedly affected in the transgenic lines under both well-watered and water-stressed conditions. For example, in well-watered roots, the relative elongation rate at 6 mm from the apex, in the basal region of deceleration, was threefold higher in the transgenic compared to the wild-type, whereas local elongation rates were decreased in the transgenic line in the apical region of acceleration (Figure 5E). Conversely, in water-stressed roots, the relative elongation rate at 3 mm from the apex, in the decelerating region of the shortened growth zone in this condition, was decreased by 63% in the transgenic compared to the wild-type (Figure 5F).

The causal interrelationship, if any, between the effects on cell production rate and cell elongation profiles is not known. One perspective to interpret these results is provided by spatial models of the regulation of cell elongation, wherein local elongation rates are determined by various positional control mechanisms (for example, gradients of hormones, certain metabolites, etc.) that may or may not be coordinated with effects on cell production (Green, 1976; Silk, 1992; Gonzalez et al., 2012; Yang et al., 2017). In this scenario, the modifications of apoplastic H_2_O_2_ in the transgenic lines would be interpreted as exerting major effects on local controls of cell elongation, with inhibitory and promotive effects occurring in different regions of the growth zone such that final cell lengths were not markedly altered. For example, local control of cell elongation could have involved effects of apoplastic H_2_O_2_ on cell wall loosening and tightening processes, and such effects could be region specific (Fry, 1998; Córdoba-Pedregosa et al., 2003; Foreman et al., 2003; Tyburski et al., 2010).

An alternative explanation is provided by the cellular model of organ growth regulation, wherein growth is determined by the number of cells produced in the meristem, each with a pre-specified capacity for elongation. In this view, as discussed by Beemster and Baskin (1998), changes in cell production rate influence the length of the growth zone by determining the number of cells that are undergoing elongation. From this perspective, the substantial effects on local relative elongation rates in the transgenic lines under both the well-watered and water-stressed conditions would be a consequence of the changes in cell production rate, rather than positionally determined controls of cell elongation processes. In other words, the increased number of cells produced from the meristem in well-watered transgenic roots need more space to expand and, consequentially, result in proportional lengthening of the growth zone, as was observed. Conversely, in the water-stressed transgenic roots, the decreased number of cells produced from the meristem would need less space to expand, resulting in a proportionally shortened growth zone, as also observed.

Spatial elongation patterns can also reflect changes in the temporal regulation of cell elongation. For example, in the well-watered and water-stressed transgenic roots, the cells could have been elongating for an increased or decreased duration of time, respectively, compared with the wild-type controls. However, the constancy of the residence time for cells to move from the end of the meristem to the distal end of the growth zone in the transgenic and wild-type lines under both well-watered and water-stressed conditions (approximately 6 h in all cases; Figure 6) indicates that temporal aspects of cell elongation control were not altered, regardless of changes in growth zone length. These results are similar to the findings of Sharp et al. (1988), who reported that the duration of cell elongation in the growth zone of the maize primary root was not impacted by varying levels of water deficit, despite the progressively smaller distances over which elongation occurred as the severity of water stress increased.

Our results cannot readily distinguish between the alternative possibilities of spatial versus cellular control as the basis of the changes in relative elongation rate profiles in the transgenic lines. Moreover, these two processes are not mutually exclusive and could occur concomitantly. However, the closely proportional changes in growth zone length to the changes in cell production rate, as well as the minor changes in overall cell elongation, are consistent with the possibility that regulation of cell production was the primary determinant of the effects of apoplastic H_2_O_2_ manipulation on root elongation under both well-watered and water-stressed conditions. Clearly, given that the overall rate of root elongation is determined by the product of cell production rate and final cell length, the changes in cell production appeared to play a key role in the effects on root elongation. This interpretation is similar to the findings of several previous studies in which organ growth rates were modified by changes in cell production, including those by Foard and Haber (1961), who demonstrated that reduced cell production by γ-ray irradiation resulted in reduced tissue elongation, by Beemster and Baskin (1998), who reported that developmental acceleration of *Arabidopsis* roots could be explained by increasing rates of cell production, and by Doerner et al. (1996), who demonstrated that overexpression of a cyclin gene in root meristems resulted in increased cell production and root elongation.

### How Does Apoplastic H_2_O_2_ Differentially Modulate Cell Production Under Well-Watered and Water-Stressed Conditions?

Maintenance of cellular redox balance, by regulating the production of ROS or antioxidant metabolites, has been shown to be important for cell cycle progression (Kerk and Feldman, 1995; Menon and Goswami, 2007; Diaz Vivancos et al., 2010a), and there is considerable evidence in both plant and animal systems that changes in cellular ROS levels can positively or negatively impact cell production (Tsukagoshi, 2016). For example, several studies have shown that low levels of exogenous ROS can lead to an increase in cell production, whereas scavenging endogenous ROS resulted in inhibition of cell production (Laurent et al., 2005; Diaz Vivancos et al., 2010b). ROS were also reported to oxidize key proteins involved in the initiation and maintenance of cell division (Menon and Goswami, 2007; Burhans and Heintz, 2009; Diaz Vivancos et al., 2010a). Conversely, there is evidence that changes in ROS metabolism can lead to inhibition of cell production. For example, *Arabidopsis* mutants deficient in the biosynthesis and reduction of glutathione were shown to exhibit cell cycle arrest and defects in meristem maintenance in roots and shoots (Vernoux et al., 2000; Reichheld et al., 2007; Yu et al., 2013). In addition, an indiscriminate increase in ROS levels, as may occur in response to prolonged or severe stress conditions, could lead to DNA damage and uncontrolled inhibition of cell production (Diaz Vivancos et al., 2010b). Interestingly, some studies indicate that the spatial distribution and balance of H_2_O_2_ and superoxide are important for regulating the transition from cell proliferation to elongation and differentiation in roots (Dunand et al., 2007; Tsukagoshi et al., 2010). Similar to the apical localization of water-stress-induced apoplastic H_2_O_2_ (Voothuluru and Sharp, 2013), apoplastic superoxide was also shown to occur preferentially in the apical region of the growth zone in both well-watered and osmotically stressed maize primary roots (Liszkay et al., 2004; Bustos et al., 2008). Accordingly, it is possible that differences in ROS balance may have played a role in the differential effects of increased H_2_O_2_ on cell production observed in well-watered and water-stressed roots in the present study.

Interestingly, together with two putative oxalate oxidases, a superoxide dismutase and an ascorbate peroxidase were also shown to increase in abundance specifically in the apical region of the growth zone of water-stressed maize primary roots in a cell wall proteomic study by Zhu et al. (2007). Therefore, superoxide and ascorbate could also be potential sources of the increase in H_2_O_2_ in the apical region. Superoxide may also interact with peroxidases to convert H_2_O_2_ to hydroxyl radicals, and can also reduce Fe^3+^ to Fe^2+^ to sustain the Fenton reaction, thereby increasing hydroxyl radical generation (Liszkay et al., 2004; Dunand et al., 2007). Plasma membrane NADPH oxidase is an important source of apoplastic superoxide (Foreman et al., 2003), and up-regulation of NADPH oxidase by both water stress and ABA treatment was reported in maize leaves (Jiang and Zhang, 2002). However, four NADPH oxidase-related sequences that were included in a transcriptomic analysis were not differentially expressed in the growth zone of water-stressed maize primary roots (Spollen et al., 2008). Collectively, these findings indicate that various processes may be involved in the modulation of apoplastic ROS in the growth zone of water-stressed roots.

However, it is not apparent from these previous studies how the increased levels of apoplastic H_2_O_2_ in the *oxalate oxidase* transgenic lines under both well-watered and water-stressed conditions may have modulated cell cycle processes occurring within the nucleus. Since there were no significant increases in cytosolic ROS in the apical region of the growth zone in the transgenic compared with the wild-type line under either condition (Supplementary Figure S2), it is unlikely that H_2_O_2_ itself was traversing the plasma membrane at significantly increased rates. Interestingly, an increase in apoplastic ROS production and perception has been implicated in cellular signaling and the regulation of nuclear gene transcription (Padmanabhan and Dinesh-Kumar, 2010; Foyer and Noctor, 2011; Shapiguzov et al., 2012; Waszczak et al., 2018). In particular, Munné-Bosch et al. (2013) proposed the involvement of a trans-plasma membrane ascorbate-based shuttle system in ROS signaling from the apoplast to the cytosol, and suggested its involvement in plant responses to multiple stressors. It is notable that several components involved in this pathway have been identified in the apical region of the growth zone of maize primary roots growing under both well-watered and water-stressed conditions. For example, Zhu et al. (2007) provided evidence for apoplastic accumulation of ascorbate peroxidase, which oxidizes ascorbate in the presence of H_2_O_2_, in the apical region of water-stressed roots. Also, plasma membrane proteomic analyses showed an increased abundance of ascorbate-dependent cytochrome b_561_ protein in the apical region of well-watered (Zhang et al., 2013) and water-stressed roots (Voothuluru et al., 2016). This protein can be oxidized by monodehydroascorbate and reduced by ascorbate, and has been implicated in trans-plasma membrane electron transport (Preger et al., 2009). There is also a significant increase in glutathione levels in the apical region of the growth zone of water-stressed roots (Kang, 2019). Whether these proteins and metabolites are involved in apoplastic ROS-mediated signaling and regulation of cell production in well-watered and water-stressed maize primary roots remains to be investigated.

### Is the Decrease in Cell Production in Water-Stressed Roots of Adaptive Advantage?

As noted in the Introduction, previous studies of water-stressed maize primary roots have shown that although local elongation rates were maintained in the apical region of the growth zone (Sharp et al., 1988; Liang et al., 1997), rates of cell production were decreased substantially (Fraser et al., 1990; Saab et al., 1992; Sacks et al., 1997). In the present study, similarly, local elongation rates were unaffected by water stress in the apical 2 mm of the growth zone in the CK44 wild-type line (compare Figures 5E and F), whereas cell production rate was substantially decreased (Table 2). These findings illustrate that the responses of cell elongation and proliferation to water stress are not coordinately regulated, and it has been speculated that decreased rates of cell production in roots (Sacks et al., 1997) and leaves (Skirycz et al., 2011; Claeys et al., 2012) may be of adaptive advantage for plants growing under conditions of limited water availability. As emphasized by Sacks et al. (1997), decreased cell production combined with maintenance of local elongation results in a tendency toward longer cells in the apical region of water-stressed compared with well-watered roots. In the present study, this effect was clearly apparent in the CK44 transgenic line; for example, at 2 mm from the apex, cell lengths were approximately 60 and 30 μm in the water-stressed and well-watered roots, respectively (Figures 5A,B). This response may facilitate symplastic translocation from the phloem to the meristematic cells because of the smaller number of plasmodesmata that have to be traversed (Bret-Harte and Silk, 1994; Sacks et al., 1997). In addition, decreased cell production contributes to the shortening of the growth zone toward the apex that occurs in water-stressed roots (Sharp et al., 1988), since fewer cells require less space for expansion. This effect is evidenced by the further truncation of the growth zone observed in the CK44 transgenic line compared with the wild-type (compare Figures 5E and F). This response could also facilitate solute and water transport to the apical region (Wiegers et al., 2009), thereby helping to promote osmotic adjustment and the maintenance of cell expansion (Voetberg and Sharp, 1991; Verslues and Sharp, 1999).

Intriguingly, however, the results of the present study do not provide evidence in support of these hypotheses. In particular, if the decrease in cell production in water-stressed roots provides an advantage for root growth maintenance, then the partial restoration of cell production that occurred in the H_2_O_2_ scavenger experiments might be expected to have resulted in inhibition of root elongation. This was not the case; instead, root elongation increased in association with the increase in cell production (Table 4). Accordingly, the results indicate that the normal decrease in cell production under water stress results in root elongation being more inhibited than would otherwise be the case, and thus the adaptive significance of the response, if any, is not clear.

## Conclusion

The combined results from the characterization of *oxalate oxidase* transgenic lines and H_2_O_2_ scavenger experiments present compelling evidence that apoplastic H_2_O_2_ positively and negatively modified primary root elongation under well-watered and water-stressed conditions, respectively. The effects of increased H_2_O_2_ on root elongation were attributable to modulation of both cell production rate and changes in the spatial profiles of cell elongation, although only minor changes in overall cell elongation occurred. The results are consistent with the possibility that the effects on cell production were the primary determinant of the effects on root elongation under both well-watered and water-stressed conditions. Future studies will explore the intra-cellular signaling mechanisms involved in apoplastic H_2_O_2_-mediated differential regulation of cell production under well-watered and water-stressed conditions.

## Data Availability Statement

All datasets generated for this study are included in the article/Supplementary Material.

## Author Contributions

PV, PM, JZ, and RS conceived and designed the experiments. JS provided the transgenic lines. PV, JZ, and MY characterized root growth responses and oxalate oxidase activity staining. I-JC and JZ conducted apoplastic ROS assays. PV conducted kinematic analyses and cytosolic ROS assays. PV, I-JC, and MO conducted transgene expression studies. PM and PV conducted H_2_O_2_ scavenger experiments. PV, MO, and RS drafted the manuscript with revisions from other authors.

## Conflict of Interest

The authors declare that the research was conducted in the absence of any personal, commercial or financial relationships that could be construed as a potential conflict of interest.
