# Supplementary material for: Apoplastic Hydrogen Peroxide in the Growth Zone of the Maize Primary Root. Increased Levels Differentially Modulate Root Elongation Under Well-Watered and Water-Stressed Conditions
Source: Front Plant Sci. 2020 Apr 21;11:392. doi: 10.3389/fpls.2020.00392 (PMC7186474; doi:10.3389/fpls.2020.00392)
Supplement: Supplementary file 2 [file Presentation_1.pptx]

## Slide 1
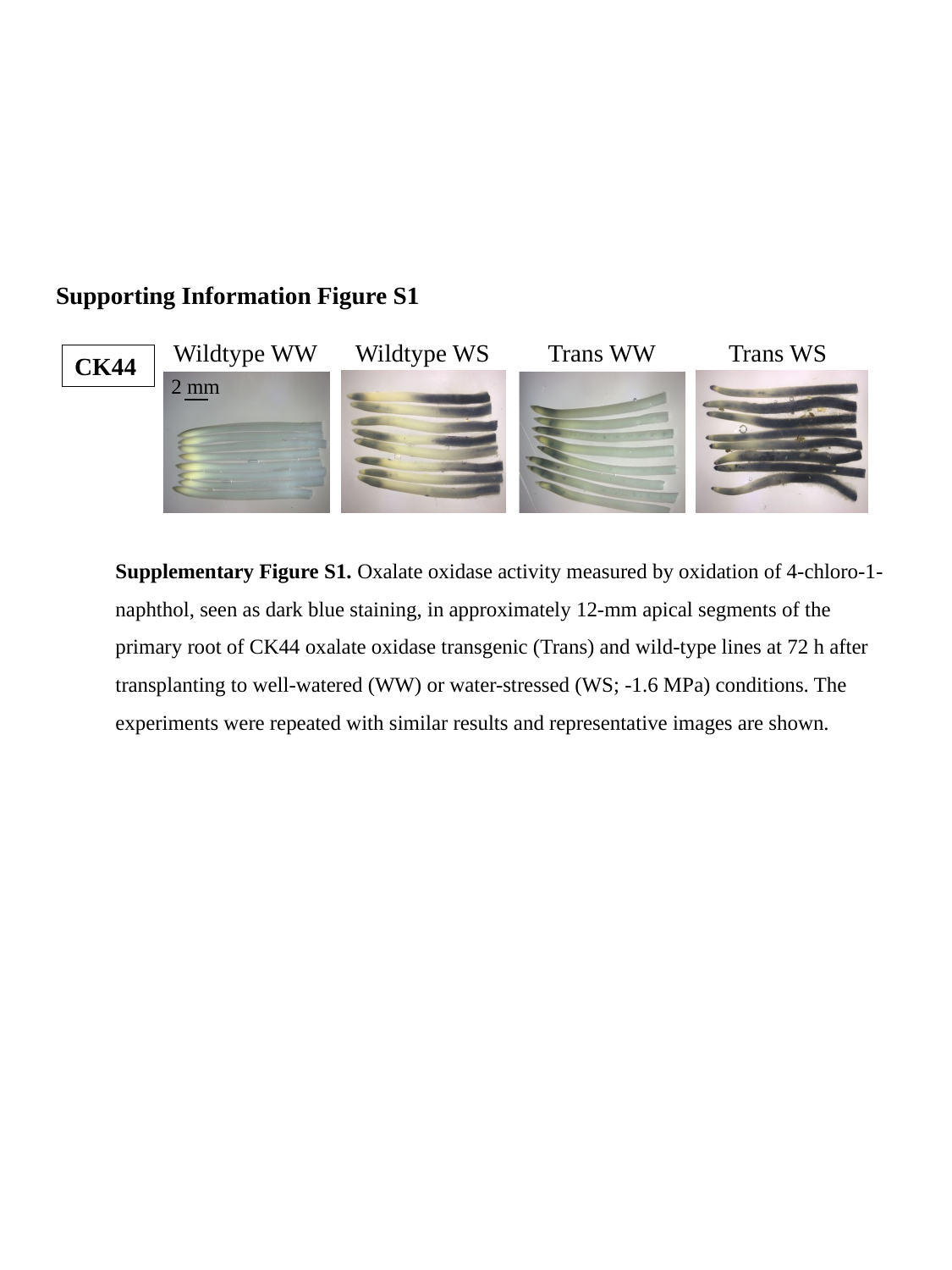

Supporting Information Figure S1
Wildtype WS
Wildtype WW
Trans WW
Trans WS
CK44
2 mm
Supplementary Figure S1. Oxalate oxidase activity measured by oxidation of 4-chloro-1-naphthol, seen as dark blue staining, in approximately 12-mm apical segments of the primary root of CK44 oxalate oxidase transgenic (Trans) and wild-type lines at 72 h after transplanting to well-watered (WW) or water-stressed (WS; -1.6 MPa) conditions. The experiments were repeated with similar results and representative images are shown.
