# Supplementary material for: Apoplastic Hydrogen Peroxide in the Growth Zone of the Maize Primary Root. Increased Levels Differentially Modulate Root Elongation Under Well-Watered and Water-Stressed Conditions
Source: Front Plant Sci. 2020 Apr 21;11:392. doi: 10.3389/fpls.2020.00392 (PMC7186474; doi:10.3389/fpls.2020.00392)
Supplement: Supplementary file 3 [file Presentation_2.pptx]

## Slide 1
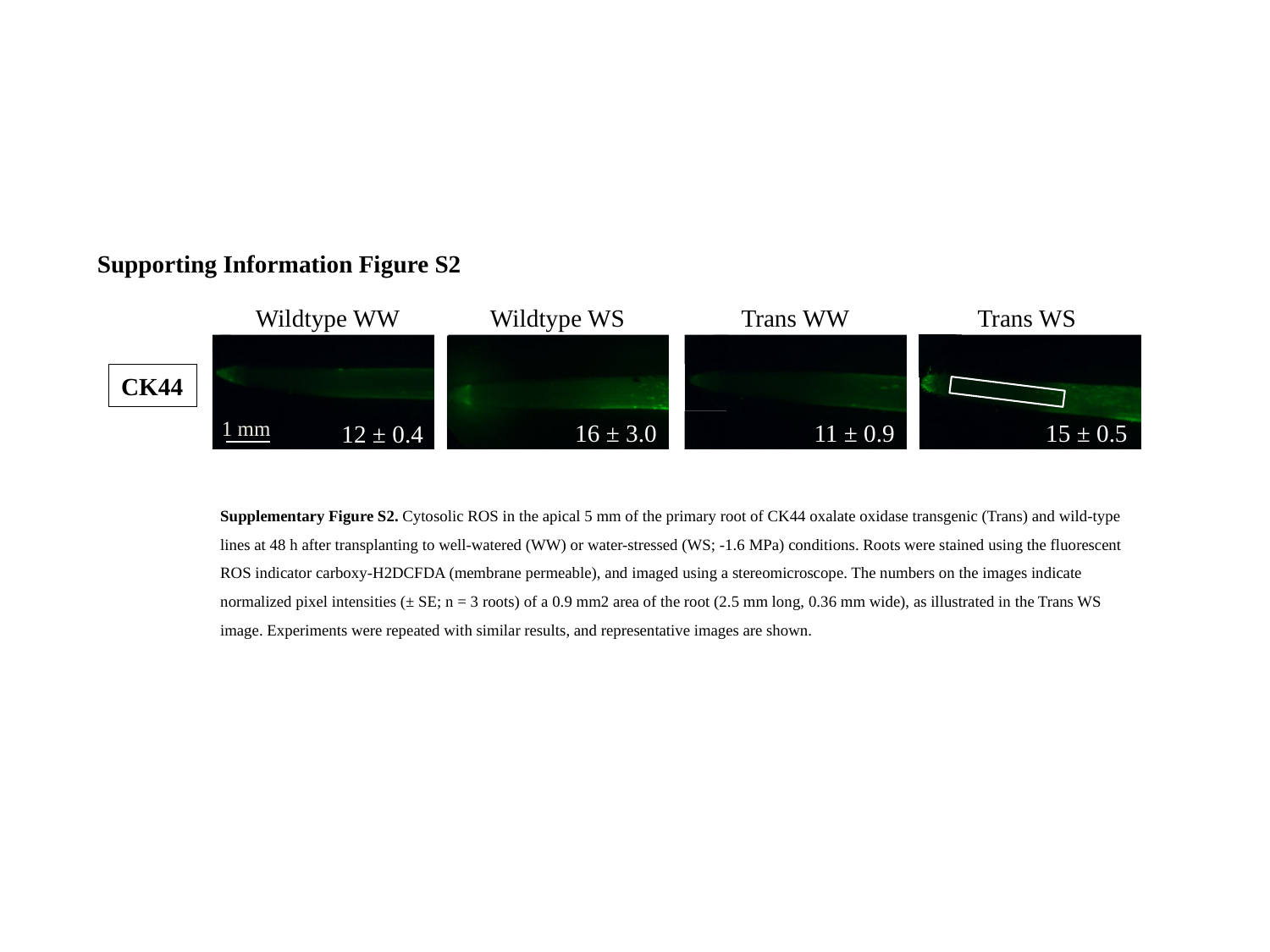

Supporting Information Figure S2
Wildtype WW
Wildtype WS
Trans WW
Trans WS
CK44
15 ± 0.5
1 mm
12 ± 0.4
11 ± 0.9
16 ± 3.0
Supplementary Figure S2. Cytosolic ROS in the apical 5 mm of the primary root of CK44 oxalate oxidase transgenic (Trans) and wild-type lines at 48 h after transplanting to well-watered (WW) or water-stressed (WS; -1.6 MPa) conditions. Roots were stained using the fluorescent ROS indicator carboxy-H2DCFDA (membrane permeable), and imaged using a stereomicroscope. The numbers on the images indicate normalized pixel intensities (± SE; n = 3 roots) of a 0.9 mm2 area of the root (2.5 mm long, 0.36 mm wide), as illustrated in the Trans WS image. Experiments were repeated with similar results, and representative images are shown.
