# Supplementary material for: Apoplastic Hydrogen Peroxide in the Growth Zone of the Maize Primary Root. Increased Levels Differentially Modulate Root Elongation Under Well-Watered and Water-Stressed Conditions
Source: Front Plant Sci. 2020 Apr 21;11:392. doi: 10.3389/fpls.2020.00392 (PMC7186474; doi:10.3389/fpls.2020.00392)
Supplement: Supplementary file 4 [file Presentation_3.pptx]

## Slide 1
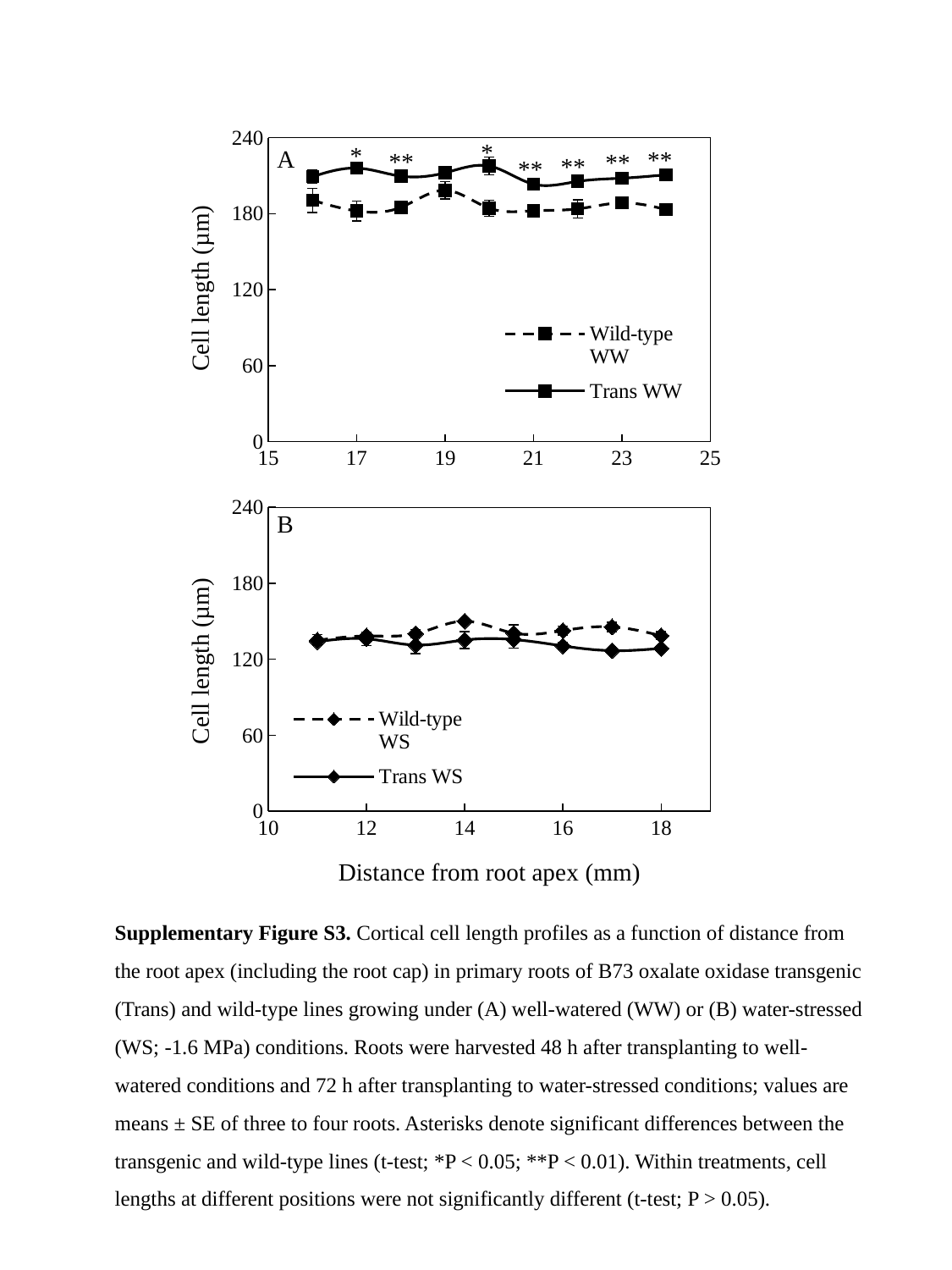

### Chart
| Category | | |
|---|---|---|*
*
A
**
**
**
**
**
Cell length (µm)
### Chart
| Category | | Trans WS |
|---|---|---|B
Cell length (µm)
Distance from root apex (mm)
Supplementary Figure S3. Cortical cell length profiles as a function of distance from the root apex (including the root cap) in primary roots of B73 oxalate oxidase transgenic (Trans) and wild-type lines growing under (A) well-watered (WW) or (B) water-stressed (WS; -1.6 MPa) conditions. Roots were harvested 48 h after transplanting to well-watered conditions and 72 h after transplanting to water-stressed conditions; values are means ± SE of three to four roots. Asterisks denote significant differences between the transgenic and wild-type lines (t-test; *P < 0.05; **P < 0.01). Within treatments, cell lengths at different positions were not significantly different (t-test; P > 0.05).
