# Supplementary material for: Apoplastic Hydrogen Peroxide in the Growth Zone of the Maize Primary Root. Increased Levels Differentially Modulate Root Elongation Under Well-Watered and Water-Stressed Conditions
Source: Front Plant Sci. 2020 Apr 21;11:392. doi: 10.3389/fpls.2020.00392 (PMC7186474; doi:10.3389/fpls.2020.00392)
Supplement: Supplementary file 5 [file Presentation_4.pptx]

## Slide 1
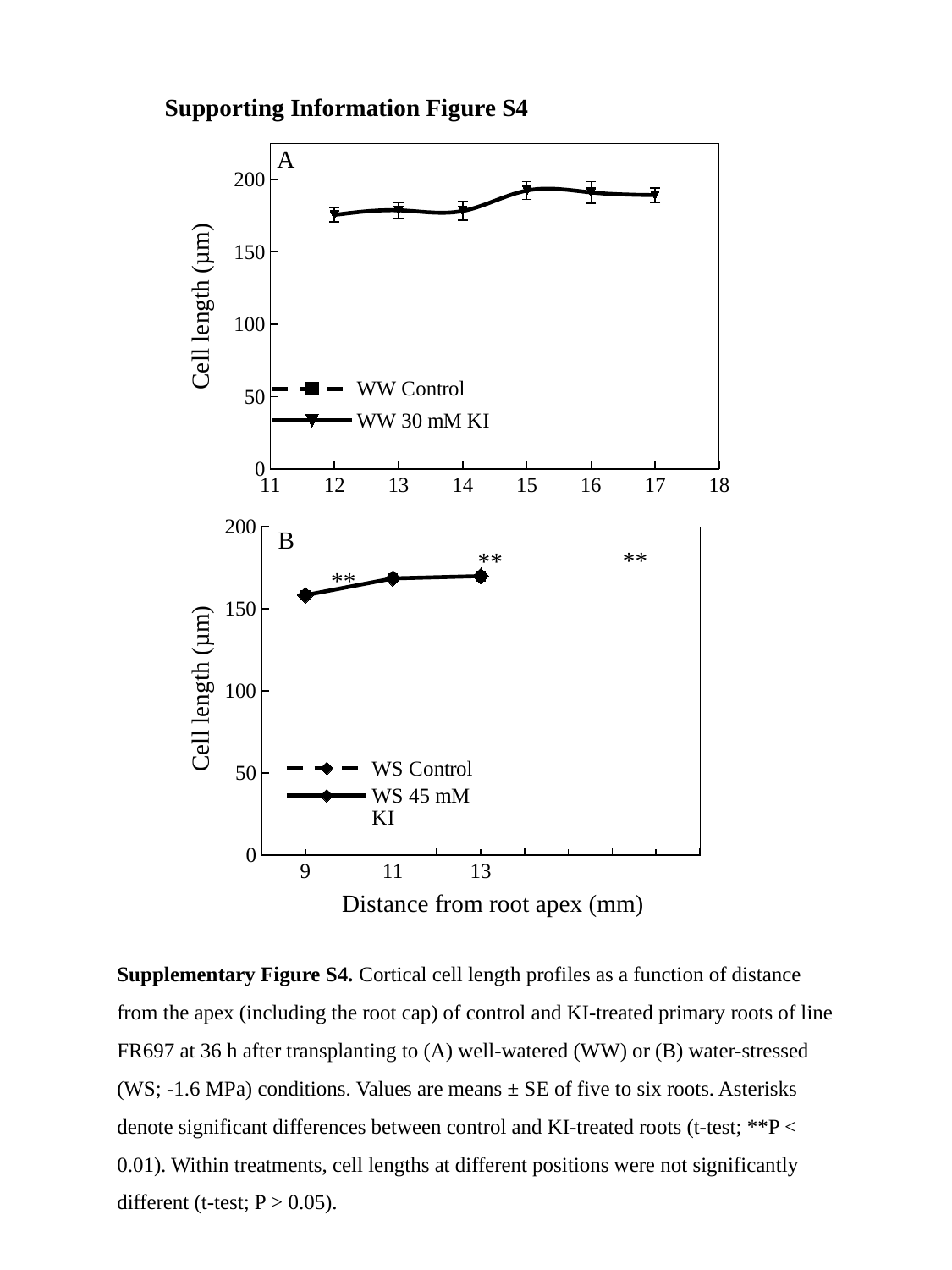

Supporting Information Figure S4
### Chart
| Category | | |
|---|---|---|A
Cell length (µm)
### Chart
| Category | WS Control | WS 45 mM KI |
|---|---|---|
| 9 | 142.75904761904758 | 158.25520710059175 |
| 11 | 141.26192307692307 | 168.48166666666654 |
| 13 | 144.9169811320754 | 169.88826086956524 |B
**
**
**
Cell length (µm)
Distance from root apex (mm)
Supplementary Figure S4. Cortical cell length profiles as a function of distance from the apex (including the root cap) of control and KI-treated primary roots of line FR697 at 36 h after transplanting to (A) well-watered (WW) or (B) water-stressed (WS; -1.6 MPa) conditions. Values are means ± SE of five to six roots. Asterisks denote significant differences between control and KI-treated roots (t-test; **P < 0.01). Within treatments, cell lengths at different positions were not significantly different (t-test; P > 0.05).
