# Supplementary material for: Apoplastic Hydrogen Peroxide in the Growth Zone of the Maize Primary Root. Increased Levels Differentially Modulate Root Elongation Under Well-Watered and Water-Stressed Conditions
Source: Front Plant Sci. 2020 Apr 21;11:392. doi: 10.3389/fpls.2020.00392 (PMC7186474; doi:10.3389/fpls.2020.00392)
Supplement: Supplementary file 6 [file Presentation_5.pptx]

## Slide 1
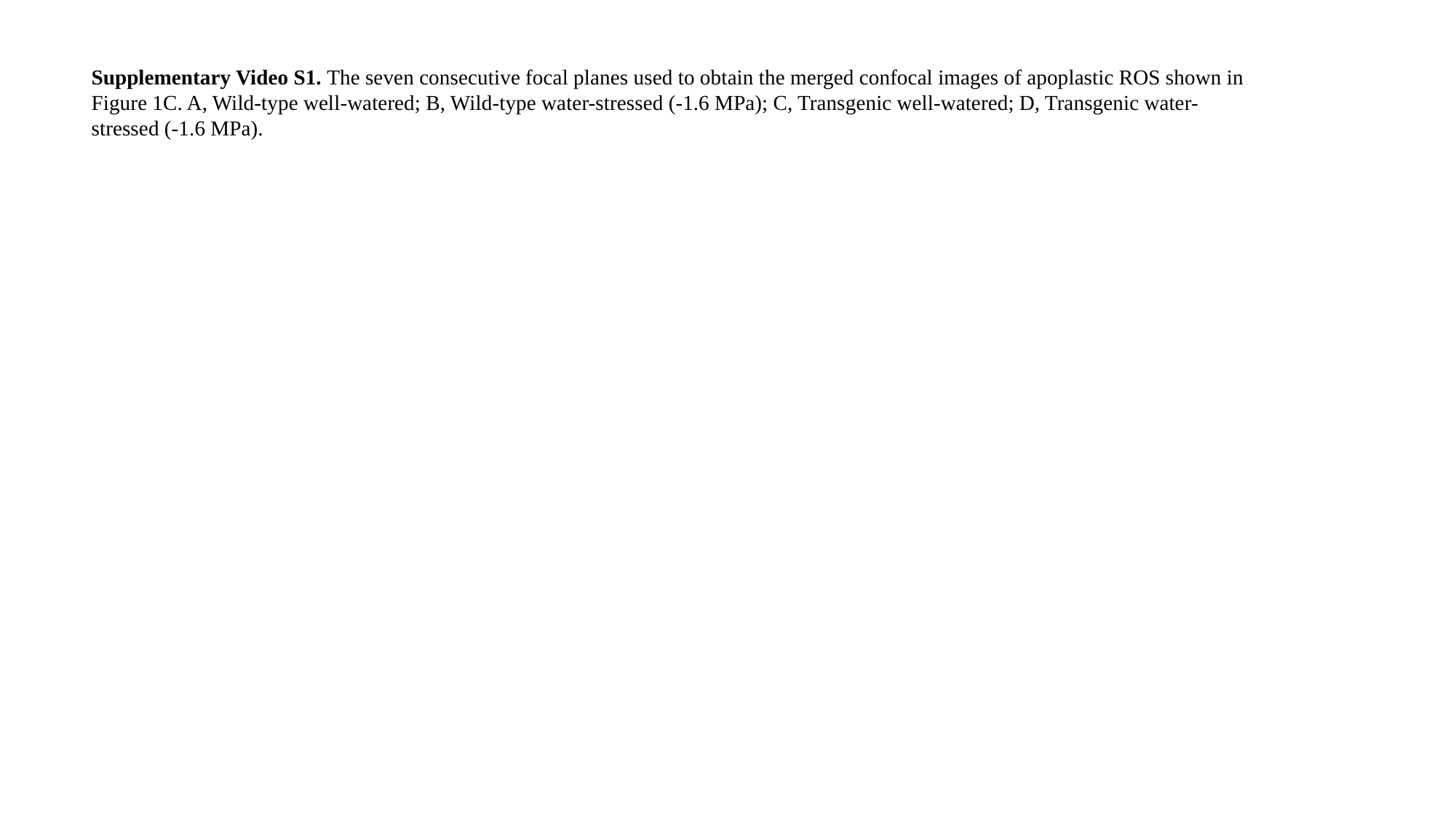

Supplementary Video S1. The seven consecutive focal planes used to obtain the merged confocal images of apoplastic ROS shown in Figure 1C. A, Wild-type well-watered; B, Wild-type water-stressed (-1.6 MPa); C, Transgenic well-watered; D, Transgenic water-stressed (-1.6 MPa).
