# Supplementary material for: Apoplastic Hydrogen Peroxide in the Growth Zone of the Maize Primary Root. Increased Levels Differentially Modulate Root Elongation Under Well-Watered and Water-Stressed Conditions
Source: Front Plant Sci. 2020 Apr 21;11:392. doi: 10.3389/fpls.2020.00392 (PMC7186474; doi:10.3389/fpls.2020.00392)
Supplement: Supplementary file 7 [file Table_1.docx]

**SUPPLEMENTARY MATERIAL**

| **Supplementary Table SI.** Primary root elongation rates of CK44 *oxalate oxidase* transgenic and wild-type lines under well-watered and water-stressed (-1.6 MPa) conditions. Elongation rates were calculated by dividing increases in root length of individual seedlings by the intervals between markings (from the experiment shown in Fig. 3A). Data are means ± SE (n = 10-20 roots). Asterisks denote significant differences between the transgenic and wild-type lines (*t*-test; **P* < 0.05; ***P* < 0.01). | | | | | | | | | |  |
| --- | --- | --- | --- | --- | --- | --- | --- | --- | --- | --- |
|  | | | | | | | | | |  |
| CK44, well-watered | | | |  | | CK44, water-stressed | | | |  |
|  | | Root elongation rate (mm h^-1^) | |  | |  | Root elongation rate (mm h^-1^) | | |  |
| Hours after transplanting | Wild-type | | Transgenic |  | Hours after transplanting | | | Wild-type | Transgenic | |
| 0-12 | 1.78 ± 0.06 | | 1.94 ± 0.03* |  |  | | |  |  | |
| 12-24 | 2.16 ± 0.04 | | 2.38 ± 0.04** |  | 0-24 | | | 0.77 ± 0.02 | 0.60 ± 0.03** | |
| 24-36 | 2.10 ± 0.04 | | 2.50 ± 0.04** |  |  | | |  |  | |
| 36-48 | 2.02 ± 0.02 | | 2.39 ± 0.05** |  | 24-48 | | | 0.86 ± 0.02 | 0.72 ± 0.02** | |
|  |  | |  |  | 48-72 | | | 1.02 ± 0.03 | 0.70 ± 0.03** | |
