# Supplementary material for: Apoplastic Hydrogen Peroxide in the Growth Zone of the Maize Primary Root. Increased Levels Differentially Modulate Root Elongation Under Well-Watered and Water-Stressed Conditions
Source: Front Plant Sci. 2020 Apr 21;11:392. doi: 10.3389/fpls.2020.00392 (PMC7186474; doi:10.3389/fpls.2020.00392)
Supplement: Supplementary file 8 [file Table_2.docx]

| **Supplementary Table S2.** Primary root elongation rates of B73 *oxalate oxidase* transgenic and wild-type lines under well-watered and water-stressed (-1.6 MPa) conditions. Elongation rates were calculated by dividing increases in root length of individual seedlings by the intervals between markings (from the experiment shown in Fig. 3B). Data are means ± SE (n = 18-32 roots). Asterisks denote significant differences between the transgenic and wild-type lines (*t*-test; **P* < 0.05; ***P* < 0.01). | | | | | | | | | | |  |
| --- | --- | --- | --- | --- | --- | --- | --- | --- | --- | --- | --- |
| B73, well-watered | | | | |  | | B73, water-stressed | | | | |
|  | | Root elongation rate (mm h^-1^) | | |  | |  | | Root elongation rate (mm h^-1^) | | |
| Hours after transplanting | Wild-type | | Transgenic |  | | Hours after transplanting | | Wild-type | | Transgenic |  |
| 0-12 | 1.84 ± 0.03 | | 2.21 ± 0.08* |  | |  | |  | |  |  |
| 12-24 | 2.01 ± 0.05 | | 2.89 ± 0.08** |  | | 0-24 | | 0.94 ± 0.03 | | 0.70 ± 0.03** |  |
| 24-36 | 2.00 ± 0.06 | | 2.62 ± 0.06** |  | |  | |  | |  |  |
| 36-48 | 1.95 ± 0.05 | | 2.58 ± 0.04** |  | | 24-48 | | 1.15 ± 0.03 | | 0.96 ± 0.03** |  |
|  |  | |  |  | | 48-72 | | 1.25 ± 0.04 | | 0.95 ± 0.06** |  |
